# Use of mixed Li/K metal TMP amide (LiNK chemistry) for the synthesis of [2.2]metacyclophanes

**DOI:** 10.3762/bjoc.7.145

**Published:** 2011-09-09

**Authors:** Marco Blangetti, Patricia Fleming, Donal F O'Shea

**Affiliations:** 1 Centre for Synthesis and Chemical Biology, School of Chemistry and Chemical Biology, University College Dublin, Belfield, Dublin 4, Ireland

**Keywords:** benzylic metalation, LiNK chemistry, [2.2]metacyclophane, oxidative coupling, planar chirality

## Abstract

A new two-step general approach to [2.2]metacyclophane synthesis from substituted *m*-xylenes is described. The strategy employs a selective benzylic metalation and oxidative C–C bond formation for both synthetic operations. Regioselective benzylic metalation is achieved using the BuLi, KO*t*-Bu, TMP(H) (2,2,6,6-tetramethylpiperidine) combination (LiNK metalation conditions) and oxidative coupling with 1,2-dibromoethane. The synthetic ease of this approach compares favourably with previously reported methods and allows for ready access to potentially useful planar chiral derivatives.

## Introduction

**Scheme 1:**
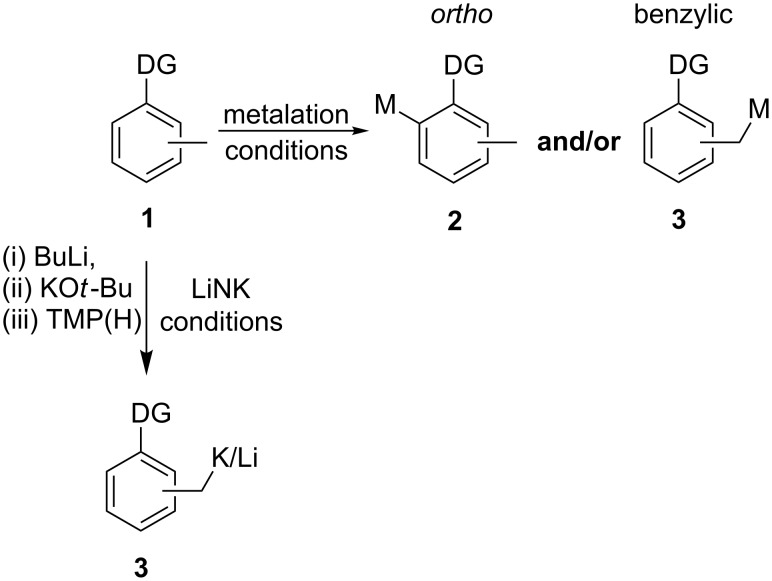
Selective benzylic metalation with LiNK conditions. DG = directing group.

While direct metalation reactions are an essential contribution to the repertoire of modern synthetic methods, an underlying and often underestimated challenge remains in the achievement of predictable selective metalations of substrates that offer several potential sites of reaction. Examples of such challenges include the selective aryl metalation of arenes containing more than one directing group (DG), arene metalation in positions not *ortho* to the directing group, or the identification of reaction conditions to achieve selective benzylic metalation of substituted toluenes **1** to provide **3** (Scheme 1) [1–5]. We recently reported that mixed Li/K metal TMP amide (LiNK metalation conditions) is uniquely suited for the selective achievement of challenging metalations. Specifically, the use of the reagent triad BuLi/KO*t*-Bu/TMP(H) to generate a mixed Li/K metal TMP amide in situ has proven to be an efficient and general method to achieve vinyl and benzylic metalations with excellent selectivity [6–7]. We now exploit this selective benzylic metalation protocol for the specific synthesis of [2.2]metacyclophanes.

Chemists have a long-standing fascination for the [2.2]cyclophane structures and have extensively studied their unusual physical and chemical properties induced by the close spatial proximity of their aryl rings [8]. Undoubtedly the most studied of the series (*ortho*, *meta* and *para*) are the [2.2]paracyclophanes, which have seen a recent resurgence of interest notably as planar chiral scaffolds for asymmetric catalysis [9–13]. Surprisingly, the [2.2]metacyclophanes, which also have the potential to be exploited as planar chiral templates, have received scant attention since the seminal reports of Schlögl in the early 1970s [14–16]. One possible explanation for this is the cumbersome methods required for their synthesis. Typical approaches have utilised Wurtz coupling, the oxidation and thermal (500–600 °C) extrusion of SO_2_ from dithia[3.3]cyclophanes or the photochemical ring contraction of diselena[3.3]cyclophanes [17–21].

**Scheme 2:**
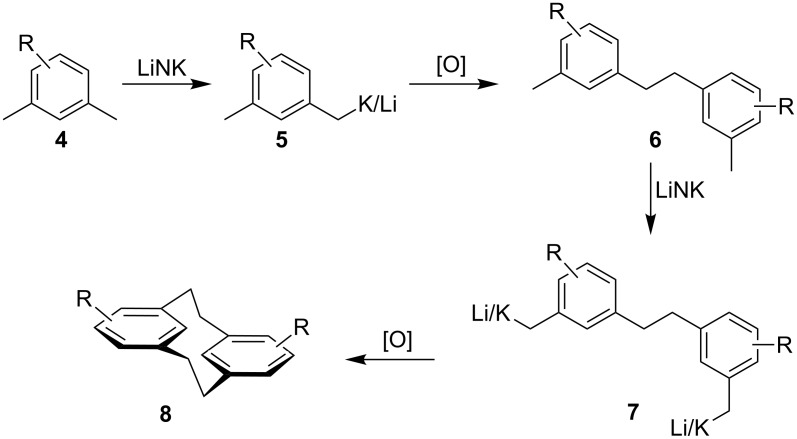
Iterative LiNK/oxidative coupling synthesis of [2.2]metacyclophanes.

We envisaged that our LiNK metalation conditions with in situ oxidative coupling could offer a facile general approach to [2.2]metacyclophanes, which would be of general synthetic interest (Scheme 2). Oxidative homo-coupling of benzyl anions has previously been noted, but it has remained relatively unexplored as a synthetic procedure [22–24]. We speculated that if oxidative coupling of the benzyl metalated xylenes **5** could be achieved to form the open dimer **6**, then a second metalation and oxidative ring closure would yield the [2.2]metacyclophanes **8**. A stepwise approach, as shown in Scheme 2, could allow for the introduction of different groups on each of the aryl rings. In addition, it could facilitate the synthesis of planar chiral derivatives without the complication of mixtures with achiral isomers being generated, which occurs if, for example, a 1-substituted-2,4-bis(halomethyl)benzene is used as the starting compound [15].

## Results and Discussion

**Figure 1:**
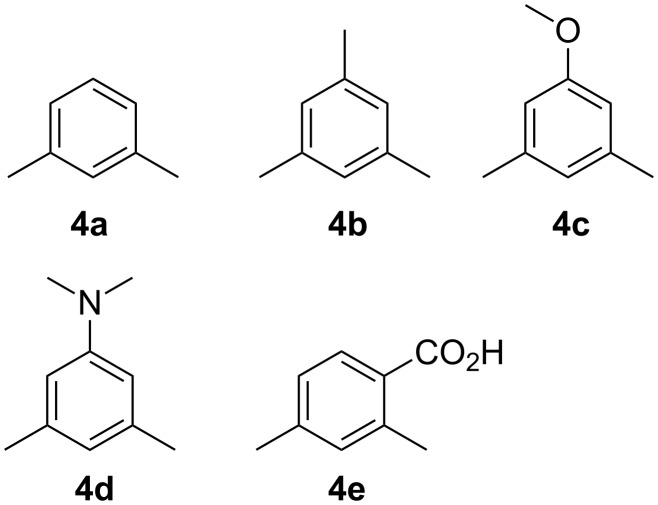
Xylene substrates.

In order to examine the scope and potential of this approach, five differently substituted xylenes were investigated, namely *m*-xylene (**4a**), mesitylene (**4b**), 1-methoxy-3,5-dimethylbenzene (**4c**), (3,5-dimethylphenyl)dimethylamine (**4d**) and 2,4-dimethylbenzoic acid (**4e**) (Figure 1). These xylenes present interesting challenges for metalation selectivity in that for derivatives **4a**–**d** both methyl groups are equivalent and so the alternative metalation sites are in the aryl ring, whereas the challenge is elevated for **4e** as it contains two differing benzylic sites and an *ortho*-aryl position primed for metalation by a strong directing group.

Sequential treatment of **4a**–**d** with BuLi, KO*t*-Bu and TMP(H) at −78 °C followed by the oxidant 1,2-dibromoethane [25] gave good to excellent yields of the targeted homo-dimer products **6a**–**d** (Table 1, entries 1–4). It was also possible to form a “mixed dimer” by the reaction of **4a** and **4c** together, which gave **6e**, containing one *m*-OCH_3_ substituted aryl ring, in a 22% yield (Table 1, entry 5) following chromatography to remove the other homo-coupled products (**6a**, **6c**). A similar approach was used in a combined reaction of **4a** and **4e**, giving purified 4-methyl-2-(3-methylphenethyl)benzoic acid (**6f**) (Table 1, entry 6). This product is the result of oxidative hetero coupling of benzylic metalated xylene and 2-(methyl-metalated)-4-methylbenzoate.

**Table 1:** Oxidative coupling of benzylic metalated xylenes **4**.

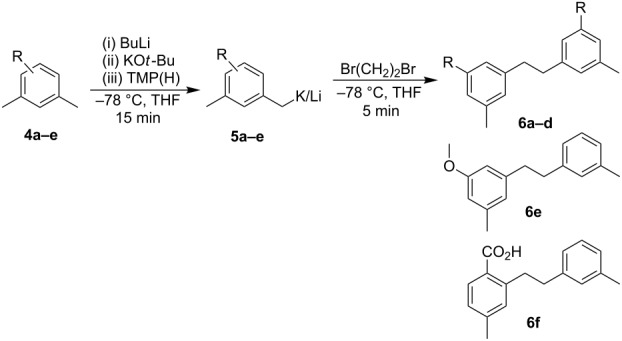				

Entry	Substrate	R	Product	Yield %

1	**4a**	H	**6a**	91
2	**4b**	CH_3_	**6b**	72
3	**4c**	OCH_3_	**6c**	92
4	**4d**	N(CH_3_)_2_	**6d**	49
5	**4a**/**4c**	OCH_3_/H	**6e**	22^a^
6	**4a**/**4e**	CO_2_H/H	**6f**	13^b^

^a^**6a** and **6c** also obtained in 14% and 49% yields respectively. ^b^**6a** and dimer of **4e** also obtained in 11% and 62% yields respectively.

**Figure 2:**
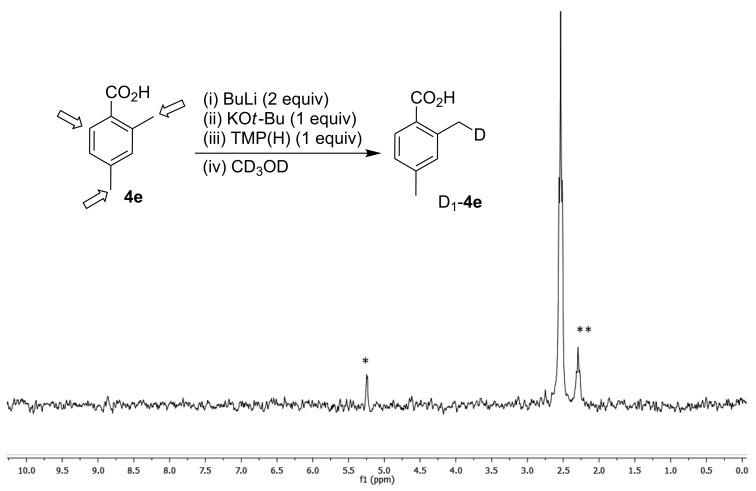
Metalation selectivity for **4e** (arrows indicate potential metalation sites)**.** ^2^H NMR spectrum in CH_2_Cl_2_. *CD_2_Cl_2_. **2-Methyl-4-D-methylbenzoic acid.

To more clearly illustrate a site selective metalation of **4e** for the methyl group *ortho* to the carboxylate, this substrate was metalated using LiNK metalation conditions (using an addition equivalent of BuLi to first deprotonate the carboxylic acid) and quenched with CD_3_OD. It was anticipated that under our low temperature yet thermodynamically controlled conditions the selective site of metalation should be the more acidic 2-methyl position [26]. This was confirmed by ^2^H NMR, which showed that incorporated deuterium was above 90% in the 2-methyl position with less than 10% in the 4-methyl group and no detectable aryl deuteration (Figure 2).

**Figure 3:**
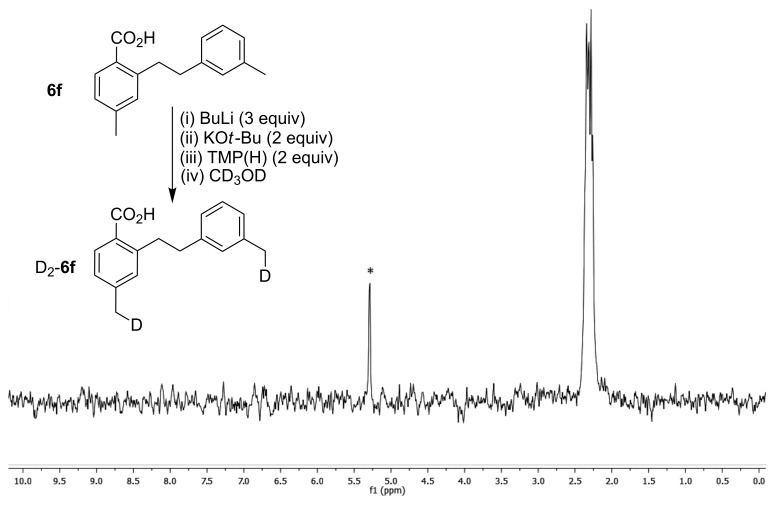
Di-metalation selectivity for **6f**. ^2^H NMR spectrum in CH_2_Cl_2_. *CD_2_Cl_2_.

A similar experiment was carried out for the even more complex hetero-dimer substrate **6f**, in which a selective di-benzylic metalation of the two different methyl positions was attempted. Treatment of **6f** with three equivalents of BuLi (one to deprotonate the carboxylic acid) and two equivalents of KO*t*-Bu/TMP(H) followed by deuteration gave the di-deuterated product D_2_-**6f**. ^2^H NMR analysis showed no aryl or bridging methylene deuteration, with deuterium incorporated only into the two non-equivalent benzylic methyl positions (Figure 3).

With the benzylic metalation confirmed, the second step to complete the [2.2]metacyclophane synthesis required identical conditions to the first to provide the di-benzylic metalated derivatives **7** (Table 2)**.** We anticipated that an intramolecular ring closing by oxidative coupling would provide the desired cyclophane product in addition to open chain oligomers or larger ring systems.

**Table 2:** Metalation/oxidative coupling to [2.2]metacyclophanes.

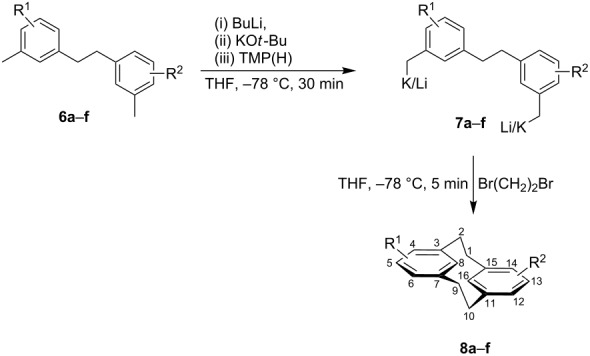					

Entry	Substrate	R^1^/R^2^	Position	Product	Yield %

1	**6a**	H/H	—	**8a**	40
2	**6b**	CH_3_/CH_3_	5,13	**8b**	54
3	**6c**	OCH_3_/OCH_3_	5,13	**8c**	33
4	**6d**	N(CH_3_)_2_/N(CH_3_)_2_	5,13	**8d**	43
5	**6e**	OCH_3_/H	5	**8e**	42
6	**6f**	CO_2_H/H	4	**8f**	39

Substrates **6a**–**e** were treated with two equivalents of BuLi/KO*t*-Bu/TMP(H) to generate the corresponding dianions **7a**–**e**, which upon oxidative coupling gave the corresponding metacyclophanes **8a**–**e**. This provided unsubstituted cyclophane **8a** in 40% yield and 5,13-disubstituted derivatives **8b**–**d**, containing CH_3_, OCH_3_ and N(CH_3_)_2_ substituents respectively, in comparable yields (Table 2, entries 1–4). In addition, the mono-methoxy substituted derivative **6e** was effectively ring closed under our reaction conditions to yield **8e** in a 42% yield. In each case, the majority of the remaining material was oligomeric in nature, although it was not characterised. Substrate **6f** offered the potential to generate the planar chiral 4-carboxylic acid substituted metacyclophane **8f**. This was readily achieved in a 39% yield, with the characteristic NMR aromatic proton signals for C(H)-8/16 observed at 4.21 and 4.18 ppm. The efficient two step synthesis of a C(4)-substituted planar chiral **8f** was achieved from inexpensive substrates, under identical reagent conditions for both steps. This compares favourably to the previously reported elaborate seven step synthesis, which was required due to the difficulties of incorporating substituents at the C(4) position after metacyclophane synthesis [27–28]. The resolution of **8f** by salt formation with (+)-1-phenylethylamine [27] has previously been accomplished.

**Figure 4:**
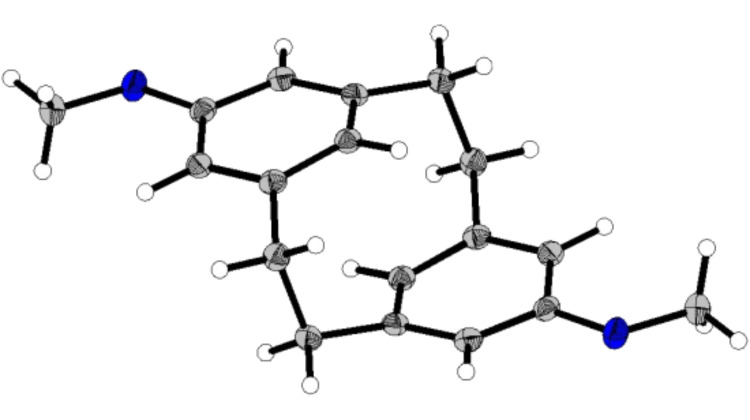
X-Ray structure of **8c** with thermal ellipsoids drawn at 50% probability level.

The stepwise *anti* conformation of the metacyclophane **8c** was confirmed by single crystal X-ray analysis. Cyclophane **8c** crystallised by the slow room temperature evaporation of a diethyl ether solution, into the monoclinic space group *P*2_1_/*n* as shown in Figure 4. **8c** contains an inversion centre with the co-planar aromatic rings bent into shallow boat forms with an angle of 8.9(1)° from planarity. The intra-annular distance as measured from C(8) to C(16) is as expected for [2.2]metacyclophanes at 2.66(1) Å [29].

## Conclusion

A new two-step general approach to [2.2]metacyclophane synthesis was described from substituted *m*-xylenes. Our strategy employs a selective benzylic metalation and oxidative C–C bond formation for both synthetic operations under LiNK metalation conditions. The synthetic ease of this approach compares favourably with previously reported methods and allows for ready access to potentially useful planar chiral derivatives. Expansion of this strategy to other chiral cyclophane architectures is ongoing and will be reported in due course. Additional synthetic applications of LiNK metalation conditions are also under development.

## Supporting Information

File 1 All experimental details, ^1^H and ^13^C NMR spectra for compounds **6a–f** and **8a–f** and X-ray crystallographic data for **8c**.
