# Supplementary material for: Use of mixed Li/K metal TMP amide (LiNK chemistry) for the synthesis of [2.2]metacyclophanes
Source: Beilstein J Org Chem. 2011 Sep 9;7:1249–54. doi: 10.3762/bjoc.7.145 (PMC3182434; doi:10.3762/bjoc.7.145)
Supplement: File 1 — All experimental details, 1H and 13C NMR spectra for compounds 6a–f and 8a–f and X-ray crystallographic data for 8c. [file Beilstein_J_Org_Chem-07-1249-s001.pdf]

Supporting Information

for

Use of mixed Li/K metal TMP amide (LiNK chemistry) for the synthesis of [2.2]metacyclophanes

*Marco Blangetti, Patricia Fleming and Donal F. O'Shea**

Centre for Synthesis and Chemical Biology, School of Chemistry and Chemical Biology,

University College Dublin, Belfield, Dublin 4, Ireland

Email: Donal F. O'Shea - donal.f.oshea@ucd.ie

* Corresponding author

All experimental details, ^{1}H and ^{13}C NMR spectra for compounds **6a-f** and **8a-f** and X-ray crystallographic data for **8c**.

Table of Contents

Experimental and references S2–S9

^{1}H and ^{13}C NMR spectra of **6a**-**6f** S10–S15

^{1}H and ^{13}C NMR spectra of **8a**-**8f** S16–S21

Synthesis and analysis of compounds D_1-**4e** and D_2-**6f** S22

^{1}H, ^{13}C and ^{2}H NMR spectra of D_1-**4e** and D_2-**6f** S23–S26

X-Ray structural data for **8c** S27–S30

Experimental

General Methods: All reactions involving air-sensitive reagents were performed under nitrogen in oven-dried glassware using syringe-septum cap technique. All solvents were purified and degassed before use. Chromatographic separations were carried out under pressure on Merck silica gel 60 using flash-column techniques. Reactions were monitored by thin-layer chromatography (TLC) carried out on 0.25 mm silica gel coated aluminium plates (60 Merck F_{254}) with UV light (254 nm) as visualizing agent. Unless specified, all reagents were used as received without further purifications. TMP(H) was distilled from CaH_2 prior to use and THF was obtained from a solvent purification system. BuLi was purchased as a 2.5 M solution in hexanes. KO*t*-Bu was purchased as a 1 M solution in THF. The exact concentration of the organolithium solution was determined by titration with diphenylacetic acid in THF prior to use [1]. ^{1}H NMR and ^{13}C NMR spectra were recorded at room temperature at 500 MHz and 125 MHz respectively and calibrated using residual undeuterated solvent as an internal reference. ^{2}H NMR (92.07 MHz) spectra were obtained in CH_2Cl_2 using residual CD_2Cl_2 as an internal standard.

1,2-Di-*m*-tolylethane (6a) [2]. A solution of *m*-xylene (**4a**) (159 mg, 1.50 mmol) in THF (15 mL) at −78 °C was treated dropwise with BuLi (2.50 M, 0.66 mL, 1.65 mmol) and stirred for 5 min. KO*t*-Bu (1.0 M in THF, 1.65 mL, 1.65 mmol) was added dropwise followed by 2,2,6,6-tetramethylpiperidine (0.25 mL, 1.50 mmol). The reaction mixture was stirred for 15 min at −78 °C, 1,2-dibromoethane (0.39 mL, 4.50 mmol) added and stirred for a further 5 min. The reaction mixture was warmed to rt and the solvent was removed under reduced pressure. Diethyl ether (30 mL) was added to the residue, washed with HCl (2 M, 3 x 10 mL), dried over sodium sulphate and concentrated to dryness. Purification by silica gel chromatography eluting with cyclohexane gave **6a** as a colourless oil (R_f = 0.70, 144 mg, 91%). ^{1}H NMR (500 MHz, $CDCl_3$) δ 7.20-7.14 (m, 2H),

7.04–6.96 (m, 6H), 2.86 (s, 4H), 2.33 (s, 6H); ^{13}C NMR (125 MHz, $CDCl_3$) δ 141.9, 137.9, 129.2, 128.2, 126.6, 125.4, 38.0, 21.4; HRMS $[M]^+$: 210.1411, $C_{16}H_{18}$ requires 210.1409.

1,2-Bis(3,5-dimethylphenyl)ethane (6b) [3]. A solution of mesitylene (**4b**) (180 mg, 1.50 mmol) in THF (15 mL) at −78 °C was treated dropwise with BuLi (2.50 M, 0.66 mL, 1.65 mmol) and stirred for 5 min. KO*t*-Bu (1.0 M in THF, 1.65 mL, 1.65 mmol) was added dropwise followed by 2,2,6,6-tetramethylpiperidine (0.25 mL, 1.50 mmol). The reaction mixture was stirred for 15 min at −78 °C, 1,2-dibromoethane (0.39 mL, 4.50 mmol) added and stirred for a further 5 min. The reaction mixture was warmed to rt and the solvent was removed under reduced pressure. Diethyl ether (30 mL) was added to the residue, washed with HCl (2 M, 3 x 10 mL), dried over sodium sulphate and concentrated to dryness. Purification by silica gel chromatography eluting with cyclohexane gave **6b** as a white solid (R_f = 0.65, 128 mg, 72%). ^{1}H NMR (500 MHz, $CDCl_3$) δ 6.85 (s, 6H), 2.81 (s, 4H), 2.30 (s, 12H); ^{13}C NMR (125 MHz, $CDCl_3$) δ 142.2, 137.9, 127.69, 127.0, 126.3, 38.2, 21.4; HRMS $[M]^+$: 238.1716, $C_{18}H_{22}$ requires 238.1722.

1,2-Bis(3-methoxy-5-methylphenyl)ethane (6c). A solution of 1-methoxy-3,5-dimethylbenzene (**4c**) (204 mg, 1.50 mmol) in THF (15 mL) at −78 °C was treated dropwise with BuLi (2.40 M, 0.69 mL, 1.65 mmol) and stirred for 5 min. KO*t*-Bu (1.0 M in THF, 1.65 mL, 1.65 mmol) was added dropwise followed by 2,2,6,6-tetramethylpiperidine (0.25 mL, 1.50 mmol). The reaction mixture was stirred for 15 min at −78 °C, 1,2-dibromoethane (0.39 mL, 4.50 mmol) added and stirred for a further 5 min. The reaction mixture was warmed to rt and the solvent was removed under reduced pressure. Diethyl ether (30 mL) was added to the residue, washed with HCl (2 M, 3 x 10 mL), dried over sodium sulphate and concentrated to dryness. Purification by silica gel chromatography eluting with 98:2 cyclohexane:EtOAc gave **6c** as a white solid (R_f = 0.50, 186 mg, 92%). ^{1}H NMR (500 MHz, $CDCl_3$) δ 6.65 (s, 2H), 6.58–6.57 (m, 4H), 3.78 (s, 6H), 2.85 (s, 4H), 2.32 (s, 6H); ^{13}C NMR (125 MHz, $CDCl_3$) δ 159.69, 143.30, 139.28, 121.75, 112.19, 111.11, 55.11, 37.94, 21.50; HRMS

$[M + Na]^+$: 293.1510, $C_{18}H_{22}O_2Na$ requires 293.1517; Analysis calcd for $C_{18}H_{22}O_2$: C, 79.96; H, 8.20. Found: C, 79.68; H, 8.26.

5,5'-(Ethane-1,2-diyl)bis(*N*,*N*,3-trimethylaniline) (6d). A solution of (3,5-dimethylphenyl)dimethylamine (**4d**) (224 mg, 1.50 mmol) in THF (15 mL) at −78 °C was treated dropwise with BuLi (2.40 M, 0.69 mL, 1.65 mmol) and stirred for 5 min. KO*t*-Bu (1.0 M in THF, 1.65 mL, 1.65 mmol) was added dropwise followed by 2,2,6,6-tetramethylpiperidine (0.25 mL, 1.50 mmol). The reaction mixture was stirred for 15 min at −78 °C, 1,2-dibromoethane (0.39 mL, 4.50 mmol) added and stirred for a further 5 min. The reaction mixture was warmed to rt and the solvent was removed under reduced pressure. Diethyl ether (30 mL) was added to the residue, washed with HCl (2 M, 3 x 10 mL), dried over sodium sulphate and concentrated to dryness. Purification by silica gel chromatography eluting with 9:1 pentane:EtOAc gave **6d** as a yellow solid (R_f = 0.40, 110 mg, 49%). ^{1}H NMR (500 MHz, $CDCl_3$) δ 6.48 (s, 2H), 6.43 (s, 2H), 6.42 (s, 2H), 2.91 (s, 12H), 2.83 (s, 4H), 2.30 (s, 6H); ^{13}C NMR (125 MHz, $CDCl_3$) δ 151.0, 143.0, 138.6, 118.1, 111.3, 110.3, 40.8, 38.7, 21.9; HRMS $[M]^+$: 296.2249, $C_{20}H_{28}N_2$ requires 296.2252; Analysis calcd for $C_{20}H_{28}N_2$: C, 81.03; H, 9.52; N, 9.45. Found: C, 80.84; H, 9.69; N, 9.19.

1-Methoxy-3-methyl-5-(3-methylphenethyl)benzene (6e). A solution of *m*-xylene (**4a**) (159 mg, 1.50 mmol) and 1-methoxy-3,5-dimethylbenzene (**4c**) (204 mg, 1.50 mmol) in THF (25 mL) at −78 °C was treated dropwise with BuLi (2.50 M, 1.20 mL, 3.00 mmol) and stirred for 5 min. KO*t*-Bu (1.0 M in THF, 3.00 mL, 3.00 mmol) was added dropwise followed by 2,2,6,6-tetramethylpiperidine (0.51 mL, 3.00 mmol). The reaction mixture was stirred for 15 min at −78 °C, 1,2-dibromoethane (0.52 mL, 6.00 mmol) added and stirred for a further 5 min. The reaction mixture was warmed to rt and the solvent was removed under reduced pressure. Diethyl ether (30 mL) was added to the residue, washed with HCl (2 M, 3 x 10 mL), dried over sodium sulphate and concentrated to dryness. Purification by silica gel chromatography eluting with 98:2 pentane:diethyl

ether gave **6e** as a colourless oil (R_f = 0.65, 79 mg, 22%). ^{1}H NMR (500 MHz, $CDCl_3$) δ 7.17 (t, *J* = 7.5 Hz, 1H), 7.05–6.98 (m, 3H), 6.63 (s, 1H), 6.56 (d, *J* = 7.5 Hz, 2H), 3.76 (s, 3H), 2.85 (br s, 4H), 2.33 (s, 3H), 2.30 (s, 3H); ^{13}C NMR (125 MHz, $CDCl_3$) δ 159.7, 143.4, 141.9, 139.3, 137.9, 129.2, 128.2, 126.6, 125.4, 121.8, 112.2, 111.1, 55.1, 38.1, 37.9, 21.5, 21.4; HRMS $[M + H]^+$: 241.1595, $C_{17}H_{21}O$ requires 241.1592.

4-Methyl-2-(3-methylphenethyl)benzoic acid (6f) [4]**.** A solution of *m*-xylene (**4a**) (159 mg, 1.50 mmol) and 2,4-dimethylbenzoic acid (**4e**) (225 mg, 1.50 mmol) in THF (25 mL) at −78 °C was treated dropwise with BuLi (2.50 M, 1.86 mL, 4.65 mmol) and stirred for 5 min. KO*t*-Bu (1.0 M in THF, 4.65 mL, 4.65 mmol) was added dropwise followed by 2,2,6,6-tetramethylpiperidine (0.51 mL, 3.00 mmol). The reaction mixture was stirred for 15 min at −78 °C, 1,2-dibromoethane (0.52 mL, 6.00 mmol) added and stirred for a further 5 min. The reaction mixture was warmed to rt and the solvent was removed under reduced pressure. Diethyl ether (30 mL) was added to the residue, washed with HCl (2 M, 3 x 10 mL), dried over sodium sulphate and concentrated to dryness. Purification by silica gel chromatography eluting with 70:30 cyclohexane:EtOAc gave **6f** as a white solid (R_f = 0.50, 47 mg, 13%). ^{1}H NMR (500 MHz, $CDCl_3$) δ 11.88 (br s, 1H), 8.01 (d, *J* = 8.0 Hz, 1H), 7.17 (t, *J* = 7.5 Hz, 1H), 7.11 (d, *J* = 8.0 Hz, 1H), 7.09–7.03 (m, 3H), 7.00 (d, *J* = 7.5 Hz, 1H), 3.32–3.25 (m, 2H), 2.92–2.85 (m, 2H), 2.37 (s, 3H), 2.32 (s, 3H); ^{13}C NMR (125 MHz, $CDCl_3$) δ 172.8, 145.2, 143.7, 142.1, 137.8, 132.3, 132.0, 129.4, 128.2, 127.0, 126.6, 125.5, 38.1, 37.2, 21.5, 21.4; HRMS $[M-H]^+$: 253.1234, $C_{17}H_{17}O_2$ requires 253.1229.

[2.2]Metacyclophane (8a) [5]**.** A solution of 1,2-di-*m*-tolylethane (**6a**) (79 mg, 0.38 mmol) in THF (15 mL) at −78 °C was treated dropwise with BuLi (2.40 M, 0.35 mL, 0.83 mmol) and stirred for 5 min. KO*t*-Bu (1.0 M in THF, 0.83 mL, 0.83 mmol) was added dropwise followed by 2,2,6,6-tetramethylpiperidine (0.13 mL, 0.75 mmol). The reaction mixture was stirred for 30 min during which time the temperature was raised to −60 °C. Afterwards 1,2-dibromoethane (97 µL, 1.13

mmol) was added, the reaction mixture was warmed to rt and the solvent was removed under reduced pressure. Diethyl ether (30 mL) was added to the residue, washed with HCl (2 M, 3 x 10 mL), dried over sodium sulphate and concentrated to dryness. Purification by silica gel chromatography eluting with 99:1 cyclohexane:EtOAc gave **8a** as a white solid (R_f = 0.80, 31 mg, 40%). ^{1}H NMR (500 MHz, $CDCl_3$) δ 7.27 (t, *J* = 7.5 Hz, 2H), 7.06 (dd, *J* = 7.5, 1.5 Hz, 4H), 4.27 (s, 2H), 3.14–3.04 (m, 4H), 2.15–2.03 (m, 4H); ^{13}C NMR (125 MHz, $CDCl_3$): δ 138.9, 136.5, 128.8, 125.4, 40.9; HRMS $[M]^+$: 208.1254, $C_{16}H_{16}$ requires 208.1252.

5,13-Dimethyl[2.2]metacyclophane (8b) [6]**.** A solution of 1,2-bis(3,5-dimethylphenyl)ethane (**6b**) (144 mg, 0.61 mmol) in THF (20 mL) at −78 °C was treated dropwise with BuLi (2.50 M, 0.48 mL, 1.22 mmol) and stirred for 5 min. KO*t*-Bu (1.0 M in THF, 1.22 mL, 1.22 mmol) was added dropwise followed by 2,2,6,6-tetramethylpiperidine (0.21 mL, 1.22 mmol). The reaction mixture was stirred for 30 min during which time the temperature was raised to −60 °C. Afterwards 1,2-dibromoethane (0.16 mL, 1.82 mmol) was added, the reaction mixture was warmed to rt and the solvent was removed under reduced pressure. Diethyl ether (30 mL) was added to the residue, washed with HCl (2 M, 3 x 10 mL), dried over sodium sulphate and concentrated to dryness. Purification by silica gel chromatography eluting with 99:1 cyclohexane:chloroform gave **8b** as a white solid (R_f = 0.70, 77 mg, 54%). ^{1}H NMR (500 MHz, $CDCl_3$) δ 6.86 (s, 4H), 4.15 (s, 2H), 3.09–2.93 (m, 4H), 2.36 (s, 6H), 2.15–2.01 (m, 4H); ^{13}C NMR (125 MHz, $CDCl_3$) δ 139.0, 138.2, 134.1, 126.1, 40.8, 21.4; HRMS $[M]^+$: 236.1570, $C_{18}H_{20}$ requires 236.1565.

5,13-Dimethoxy[2.2]metacyclophane (8c) [7]**.** A solution of 1,2-bis(3-methoxy-5-methylphenyl)ethane (**6c**) (197 mg, 0.73 mmol) in THF (30 mL) at −78 °C was treated dropwise with BuLi (2.50 M, 0.64 mL, 1.61 mmol) and stirred for 5 min. KO*t*-Bu (1.0 M in THF, 1.61 mL, 1.61 mmol) was added dropwise followed by 2,2,6,6-tetramethylpiperidine (0.25 mL, 1.46 mmol). The reaction mixture was stirred for 30 min during which time the temperature was raised to −60

°C. Afterwards 1,2-dibromoethane (0.19 mL, 2.19 mmol) was added, the reaction mixture was warmed to rt and the solvent was removed under reduced pressure. Diethyl ether (30 mL) was added to the residue, washed with HCl (2 M, 3 x 10 mL), dried over sodium sulphate and concentrated to dryness. Purification by silica gel chromatography eluting with 94:6 pentane:EtOAc gave **8c** as a white solid (R_f = 0.70, 65 mg, 33%). ^{1}H NMR (500 MHz, $CDCl_3$) δ 6.62 (s, 4H), 4.08 (s, 2H), 3.83 (s, 6H), 3.06–2.96 (m, 4H), 2.18–2.08 (m, 4H); ^{13}C NMR (125 MHz, $CDCl_3$) δ 160.5, 140.4, 129.5, 110.8, 55.3, 41.0; HRMS $[M]^+$: 268.1460, $C_{18}H_{20}O_2$ requires 268.1463. A slow evaporation of a diethyl ether solution gave crystals suitable for X-ray structural analysis [8].

5,13-Bis(dimethylamino)[2.2]metacyclophane (8d) [9]**.** A solution of 5,5'-(ethane-1,2-diyl)bis(*N*,*N*,3-trimethylaniline) (**6d**) (110 mg, 0.37 mmol) in THF (15 mL) at −78 °C was treated dropwise with BuLi (2.50 M, 0.33 mL, 0.82 mmol) and stirred for 5 min. KO*t*-Bu (1.0 M in THF, 0.82 mL, 0.82 mmol) was added dropwise followed by 2,2,6,6-tetramethylpiperidine (0.14 mL, 0.82 mmol). The reaction mixture was stirred for 30 min during which time the temperature was raised to −60 °C. Afterwards 1,2-dibromoethane (0.10 mL, 1.15 mmol) was added, the reaction mixture was warmed to rt and the solvent was removed under reduced pressure. Diethyl ether (30 mL) was added to the residue, washed with HCl (2 M, 3 x 10 mL), dried over sodium sulphate and concentrated to dryness. Purification by crystallization from methanol gave **8d** as a pale yellow solid (47 mg, 43%). ^{1}H NMR (500 MHz, $CDCl_3$) δ 6.46 (s, 4H), 4.04 (s, 2H), 3.01–2.90 (m, 4H) superimposed to 2.95 (s, 12H), 2.22–2.04 (m, 4H); ^{13}C NMR (125 MHz, $CDCl_3$) δ 151.6, 140.4, 126.4, 110.0, 41.3, 41.1; HRMS $[M + H]^+$: 295.2169, $C_{20}H_{27}N_2$ requires 295.2174.

5-Methoxy[2.2]metacyclophane (8e) [10]**.** A solution of 1-methoxy-3-methyl-5-(3-methylphenethyl)benzene (**6e**) (79 mg, 0.33 mmol) in THF (15 mL) at −78 °C was treated dropwise with BuLi (2.50 M, 0.29 mL, 0.73 mmol) and stirred for 5 min. KO*t*-Bu (1.0 M in THF, 0.73 mL, 0.73 mmol) was added dropwise followed by 2,2,6,6-tetramethylpiperidine (0.11 mL, 0.66 mmol).

The reaction mixture was stirred for 30 min during which time the temperature was raised to −60 °C. Afterwards 1,2-dibromoethane (85 µL, 0.99 mmol) was added, the reaction mixture was warmed to rt and the solvent was removed under reduced pressure. Diethyl ether (30 mL) was added to the residue, washed with HCl (2 M, 3 x 10 mL), dried over sodium sulphate and concentrated to dryness. Purification by silica gel chromatography eluting with 99:1 cyclohexane:diethyl ether gave **8e** as a white solid (R_f = 0.55, 33 mg, 42%). ^{1}H NMR (500 MHz, $CDCl_3$) δ 7.25 (t, *J* = 7.4 Hz, 1H), 7.03 (dd, *J* = 7.4, 1.4 Hz, 2H), 6.64 (d, *J* = 0.9 Hz, 2H), 4.38 (s, 1H), 3.98 (s, 1H), 3.84 (s, 3H), 3.05 (m, 4H), 2.12 (m, 4H); ^{13}C NMR (125 MHz, $CDCl_3$) δ 160.7, 140.4, 138.9, 136.2, 129.9, 128.5, 125.3, 110.8, 55.3, 41.1, 40.9; HRMS $[M]^+$: 238.1359, $C_{17}H_{18}O$ requires 238.1358.

[2.2]Metacyclophane-4-carboxylic acid (8f) [11]**.** A solution of 4-methyl-2-(3-methylphenethyl)benzoic acid (**6f**) (31 mg, 0.12 mmol) in THF (10 mL) at −78 °C was treated dropwise with BuLi (2.50 M, 0.15 mL, 0.37 mmol) and stirred for 5 min. KO*t*-Bu (1.0 M in THF, 0.37 mL, 0.37 mmol) was added dropwise followed by 2,2,6,6-tetramethylpiperidine (42 µL, 0.25 mmol). The reaction mixture was stirred for 30 min during which time the temperature was raised to −60 °C. Afterwards 1,2-dibromoethane (32 µL, 0.37 mmol) was added, the reaction mixture was warmed to rt and the solvent was removed under reduced pressure. Diethyl ether (30 mL) was added to the residue, washed with HCl (2 M, 3 x 10 mL), dried over sodium sulphate and concentrated to dryness. Purification by silica gel chromatography eluting with 1:1 cyclohexane:diethyl ether gave **8f** as a white solid (R_f = 0.55, 12 mg, 39%). ^{1}H NMR (500 MHz, CD_3OD) δ 7.83 (d, *J* = 7.9 Hz, 1H), 7.21 (t, *J* = 7.5 Hz, 1H), 7.06 (dd, *J* = 7.9, 1.4 Hz, 1H), 7.06–6.92 (m, 2H), 4.21 (s, 1H), 4.18 (s, 1H), 4.15 (dt, *J* = 11.9, 3.6 Hz, 1H), 3.09–3.04 (m, 2H), 3.01 (dt, *J* = 12.2, 3.6 Hz, 1H), 2.10 (td, *J* = 12.2, 3.1 Hz, 1H), 2.04–1.93 (m, 2H), 1.70 (td, *J* = 11.9, 3.1 Hz, 1H); ^{13}C NMR (125 MHz, $CDCl_3$) δ 172.8, 144.5, 141.5, 139.3, 138.7, 138.1, 135.8, 132.2, 129.3, 125.8, 125.6, 125.3, 41.0, 40.7, 40.3, 39.2; HRMS $[M-H]^+$: 251.1078, $C_{17}H_{15}O_2$ requires 251.1072.

References

1. Kofron, W. G.; Baclawski, L. M. *J. Org. Chem.* **1976**, *41*, 1879–1880.

2. Smith, K; Hou, D. *J. Chem. Soc. Perkin Trans. 1* **1995**, 185–186.

3. Effenberger, F.; Kottmann, H. *Tetrahedron* **1985**, *41*, 4171–4182.

4. Burbiel, J. C. *ARKIVOC* **2006**, *13*, 16–21.

5. Wilson, D. J.; Boekelheide, V. R.; Grifinn, Jr., W. *J. Am. Chem. Soc.* **1960**, *82*, 6302–6304.

6. Shizuka, H.; Sorimachi, K; Morita, T.; Nishiyama, K.; Sato, T. *Bull. Chem. Soc. Jpn.* **1971**, *44*, 1983–1984.

7. Bodwell, G.J.; Houghton, T.J; Kennedy, J.; Mannion, M. R. *Angew. Chem., Int. Ed.* **1996**, *35*, 2121–2123.

8. Crystal structure data has been deposited at the Cambridge Crystallographic Data Centre with deposit number CCDC 825095.

9. Ueda, N.; Natsume, B.; Yanagiuchi, K.; Sakata, Y.; Enoki, T.; Saito, G.; Inokuchi, H.; Misumi, S. *Bull. Chem. Soc. Jpn.* **1983**, *56*, 775–779.

10. Sherrod, S. A.; Da Costa, R. L. *Tetrahedron Lett.* **1973**, *23*, 2083–2086.

11. Kainradl, B.; Langer, E.; Lehner, H.; Schlögl, K. *Liebigs Ann. Chem.* **1972**, *766*, 16–31.

1,2-Di-*meta*-tolylethane (6a).

^{1}H NMR

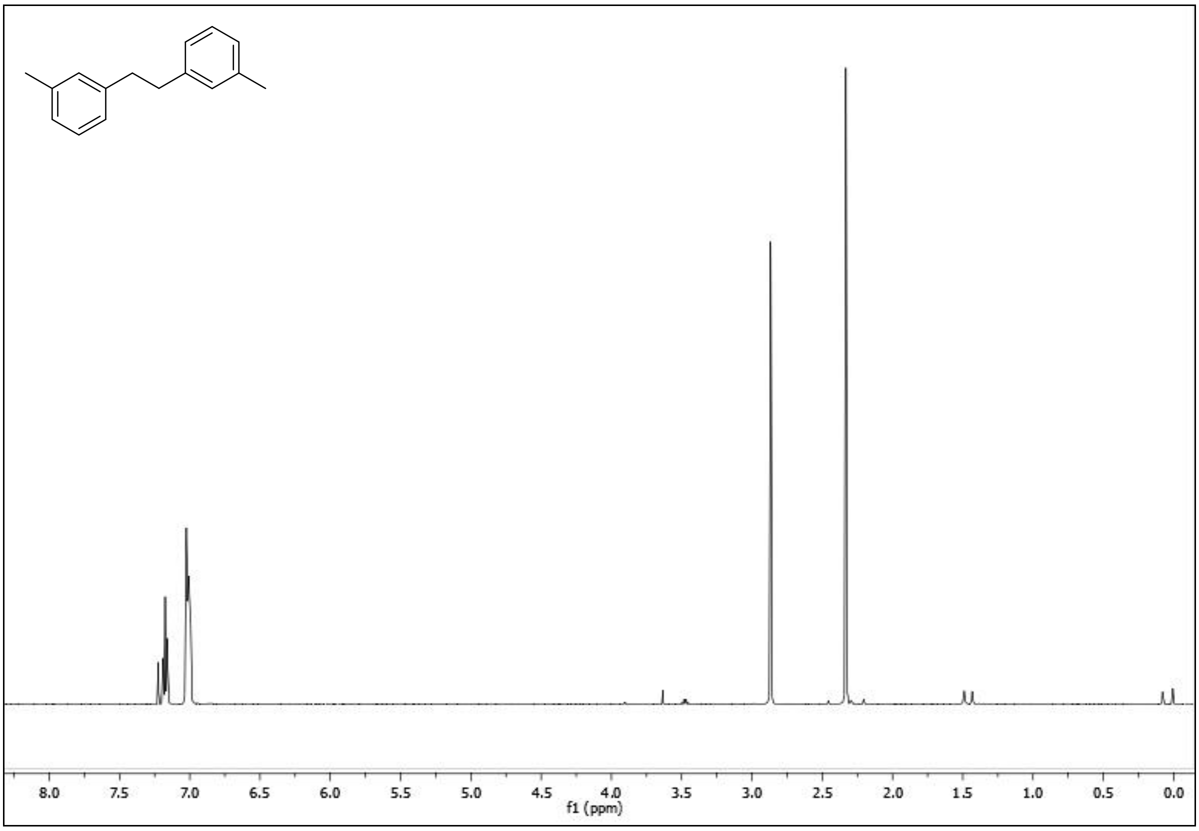

^{13}C NMR

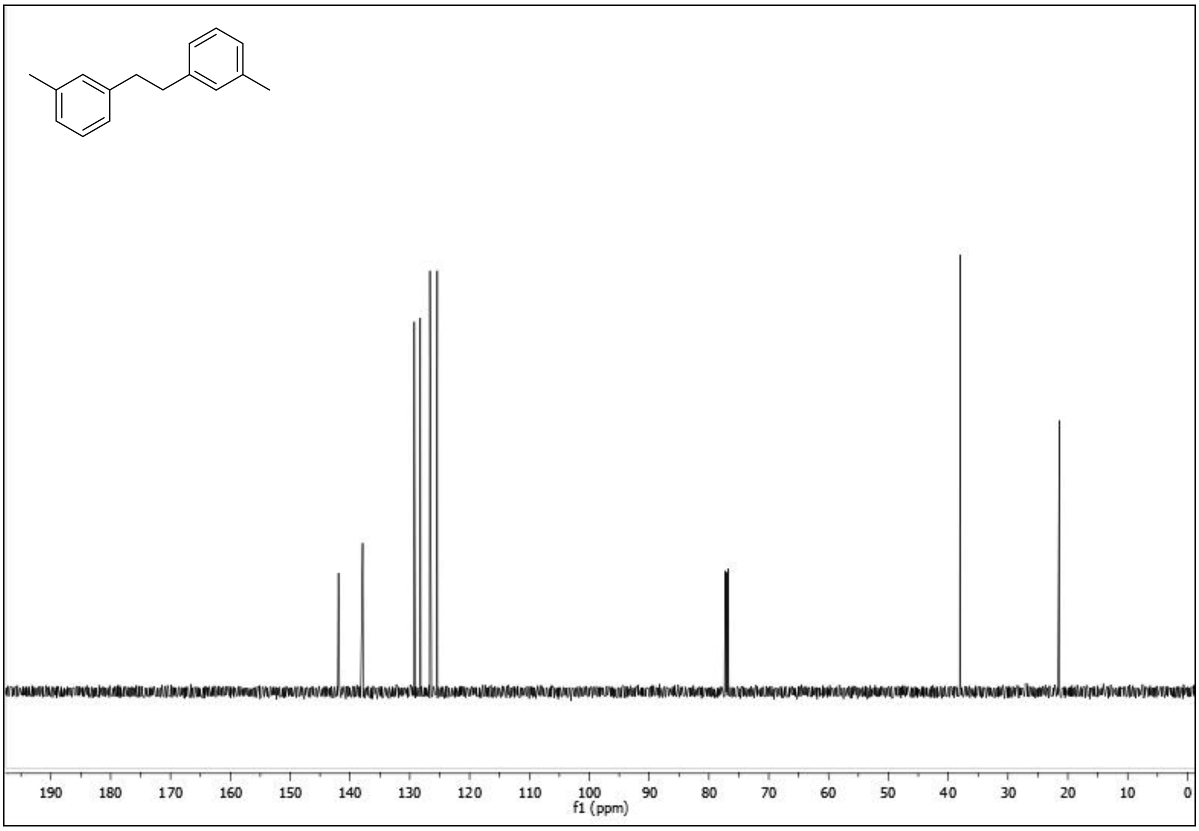

1,2-Bis(3,5-dimethylphenyl)ethane (6b).

^{1}H NMR

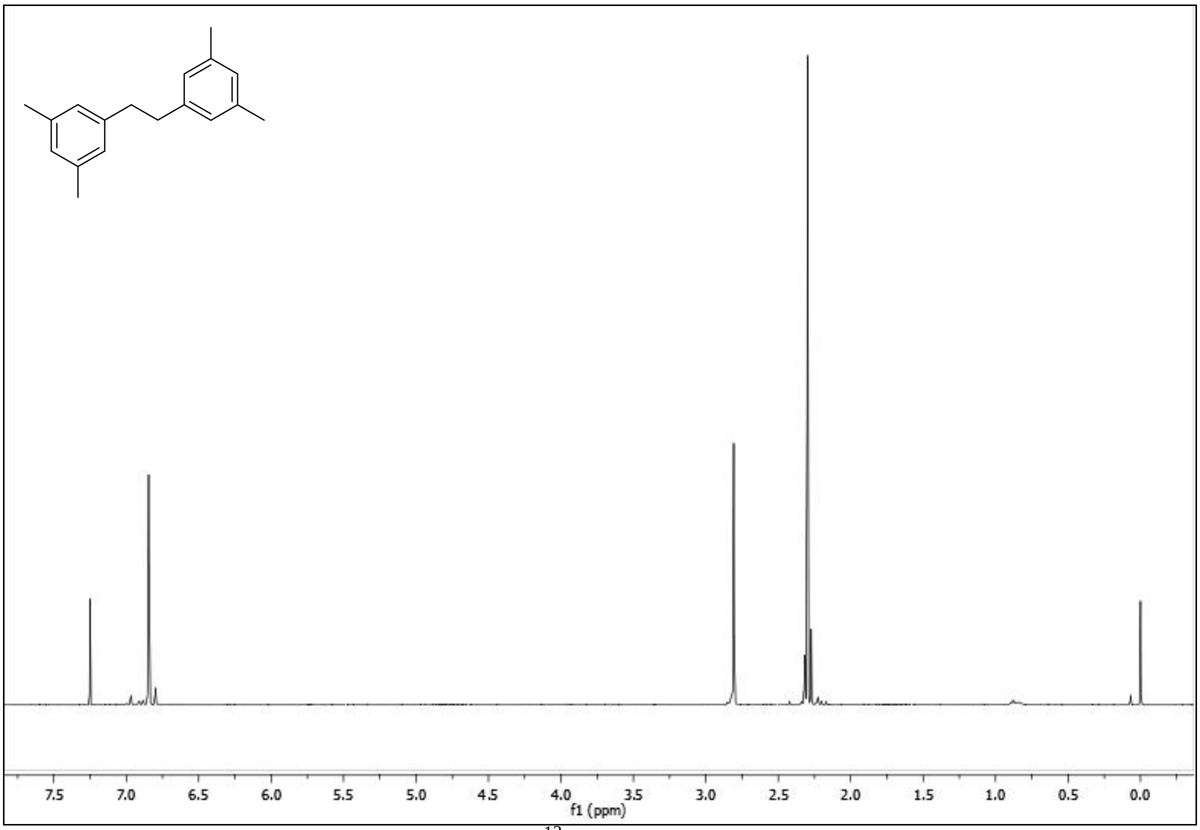

^{13}C NMR

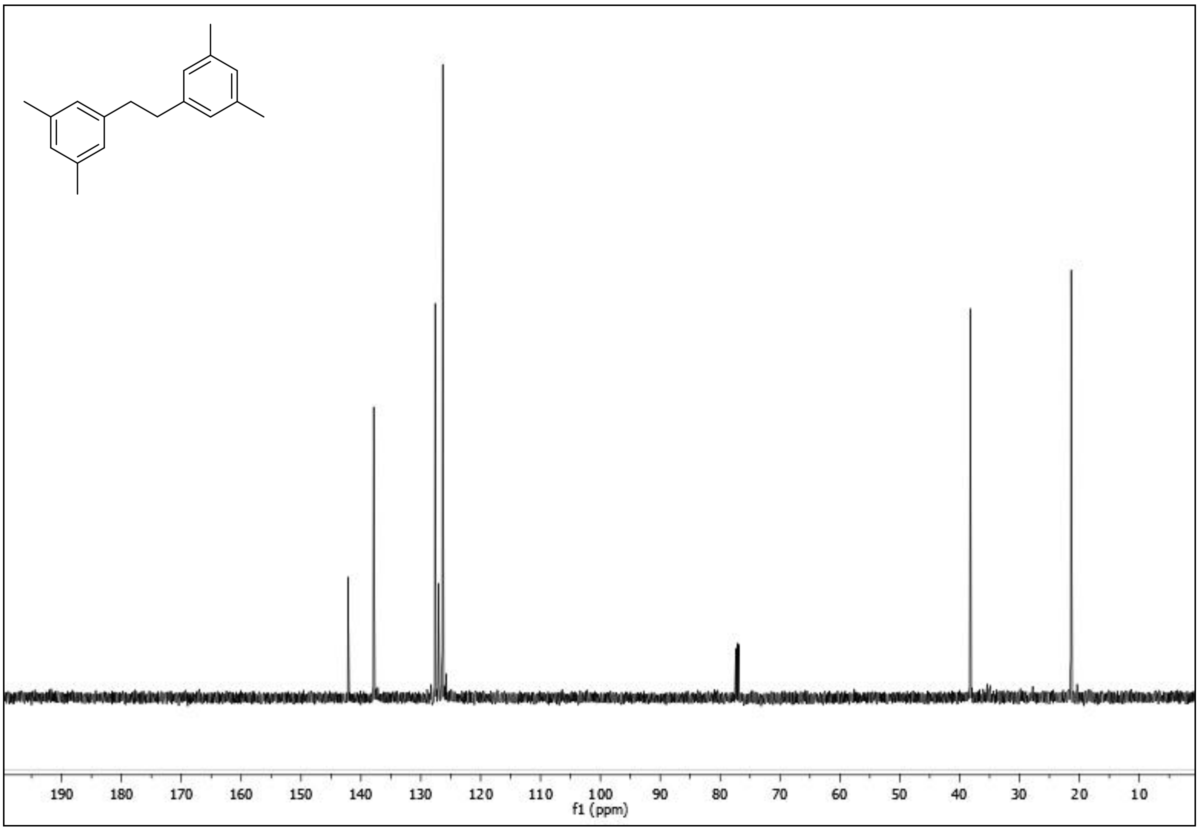

1,2-Bis(3-methoxy-5-methylphenyl)ethane (6c).

^{1}H NMR

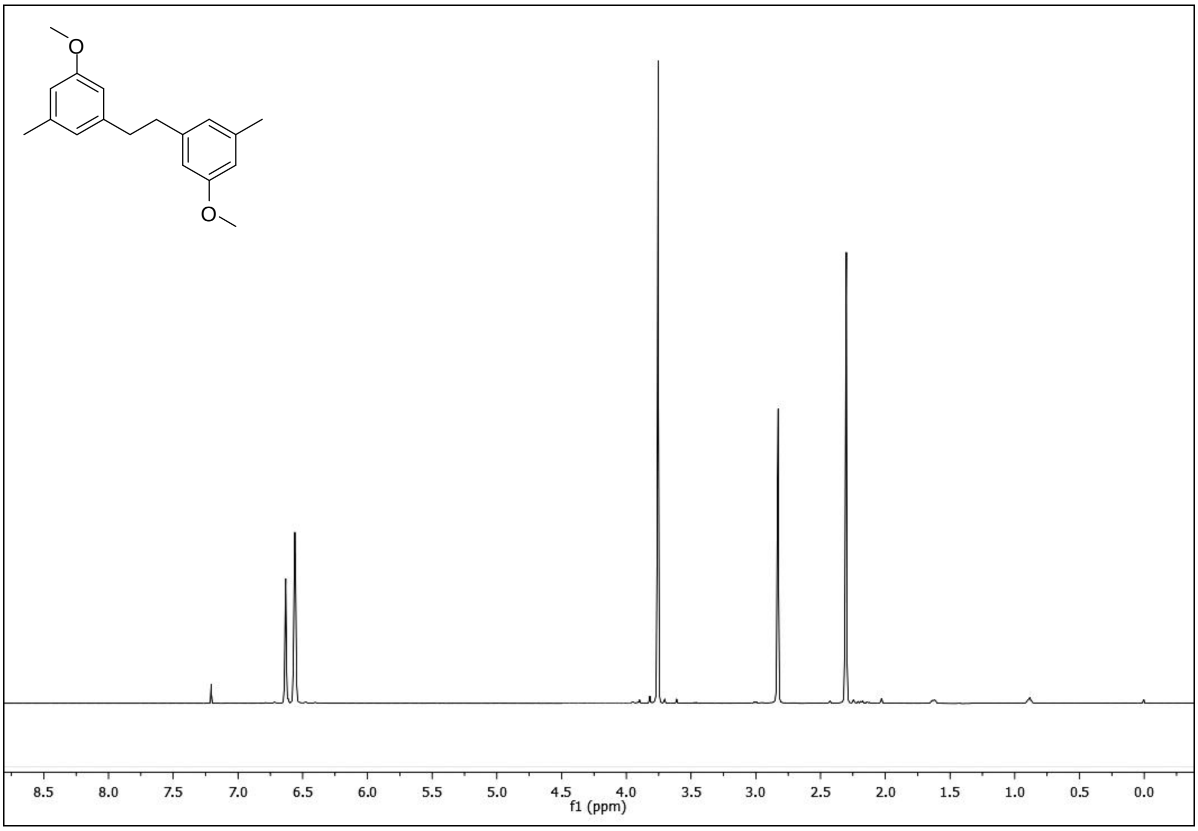

^{13}C NMR

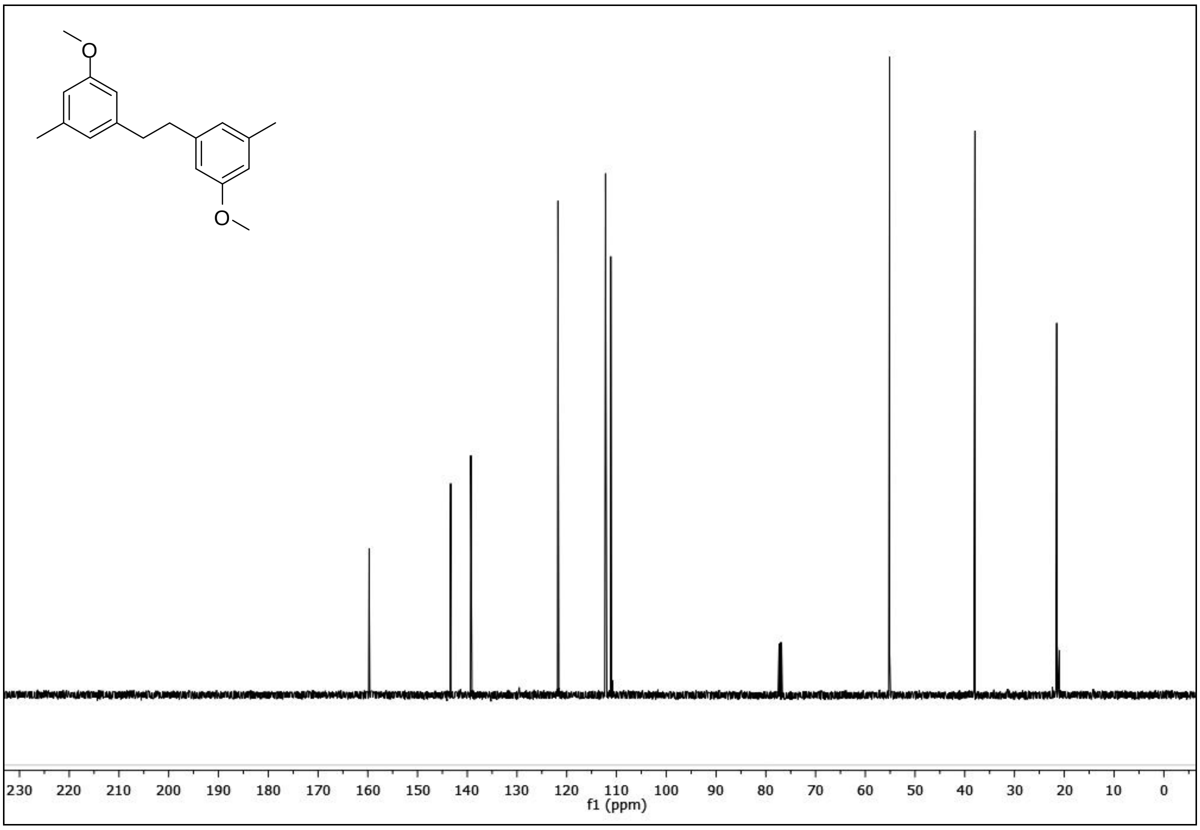

5,5'-(Ethane-1,2-diyl)bis(*N*,*N*,3-trimethylaniline) (6d).

^{1}H NMR

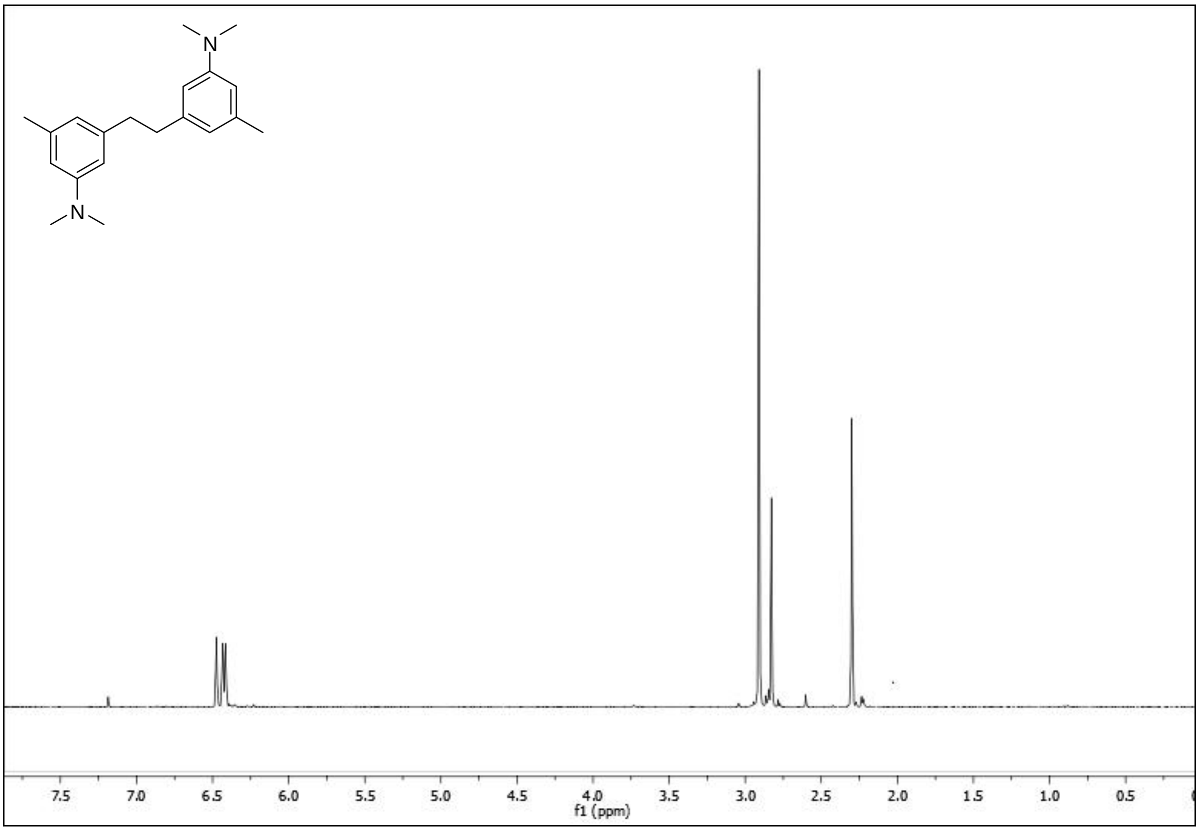

^{13}C NMR

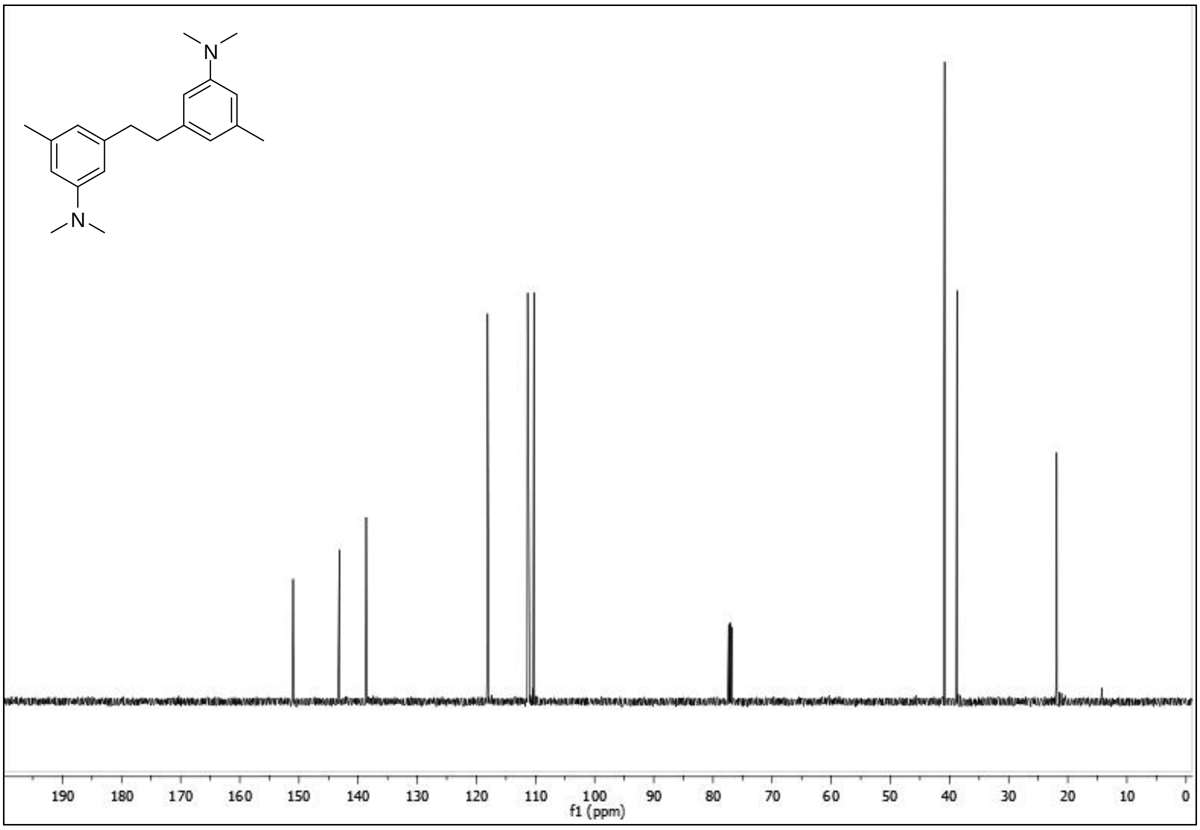

1-Methoxy-3-methyl-5-(3-methylphenethyl)benzene (6e).

^{1}H NMR

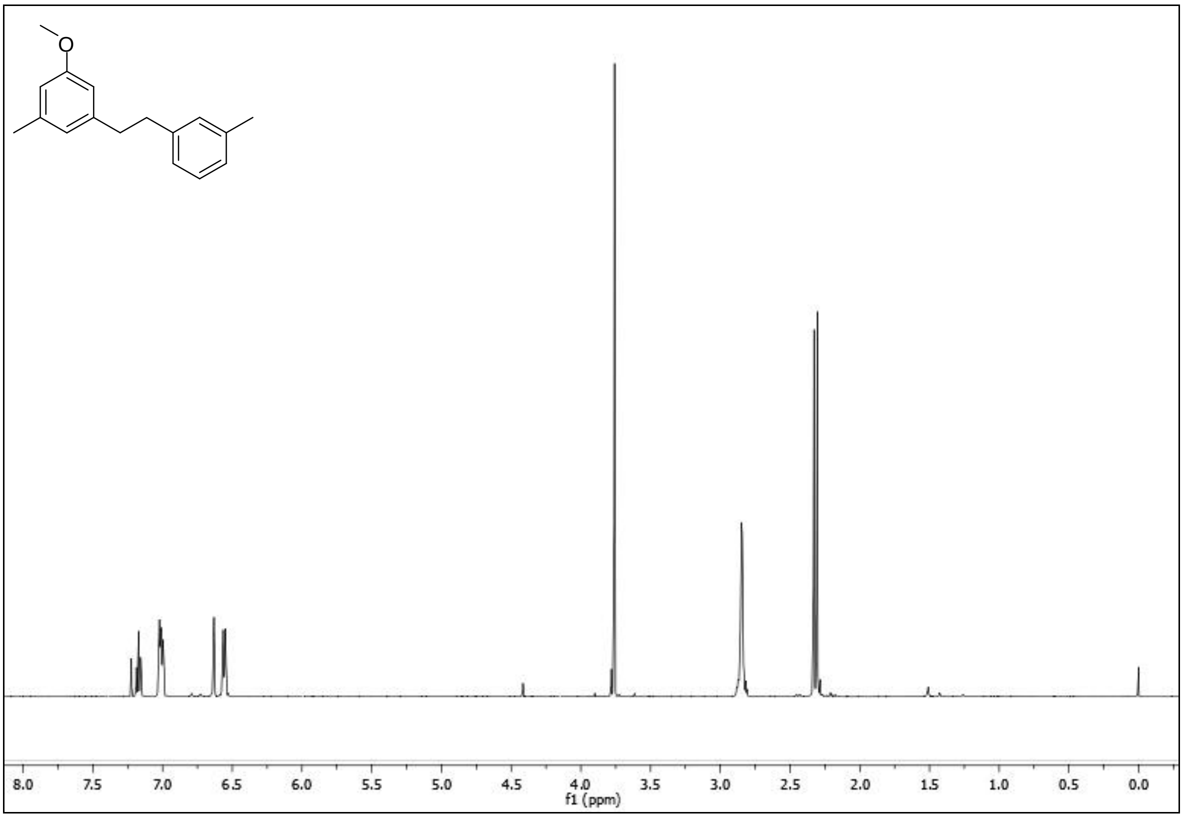

^{13}C NMR

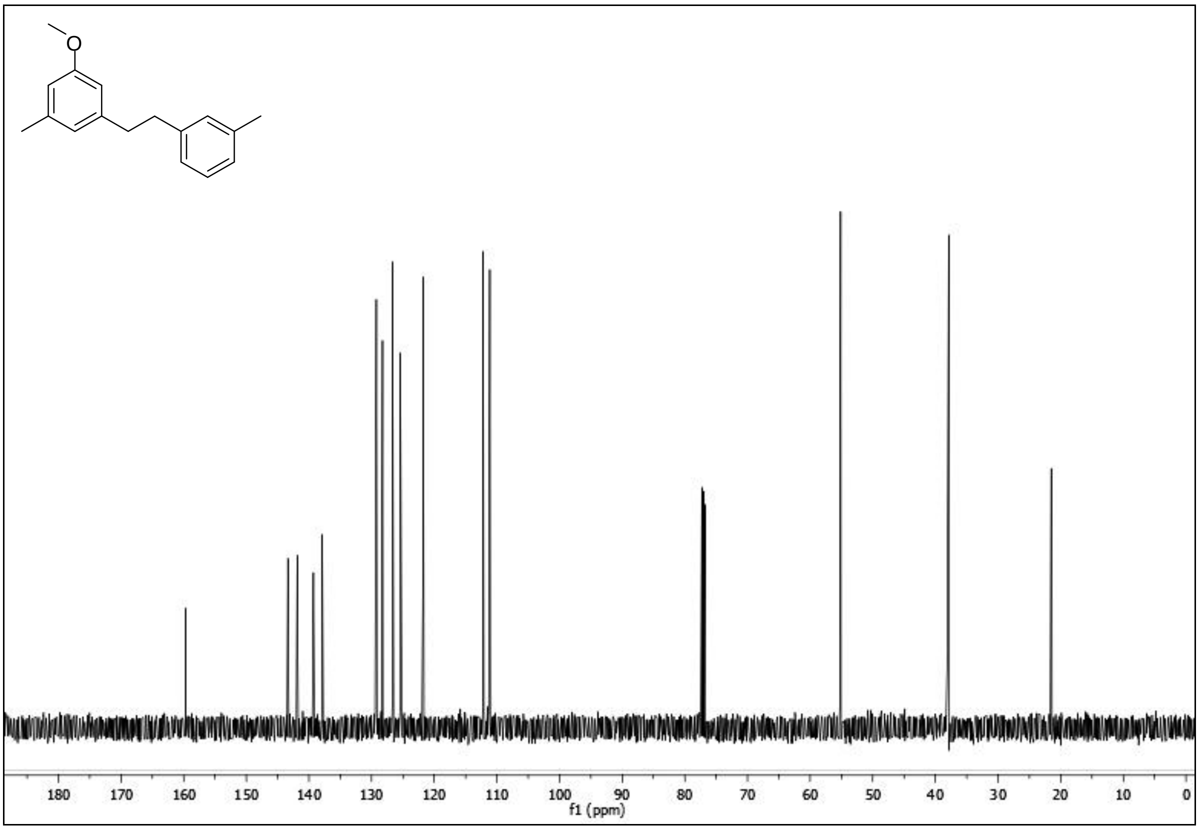

4-Methyl-2-(3-methylphenethyl)benzoic acid (6f).

^{1}H NMR

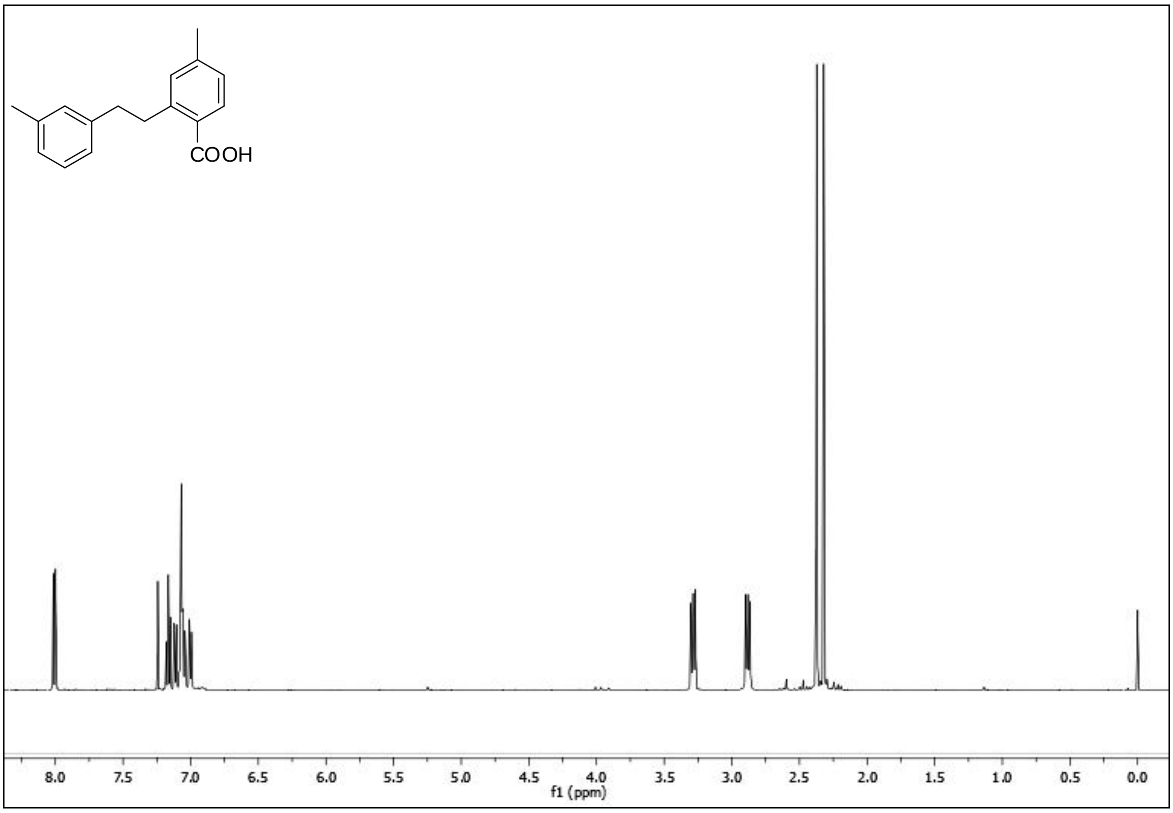

^{13}C NMR

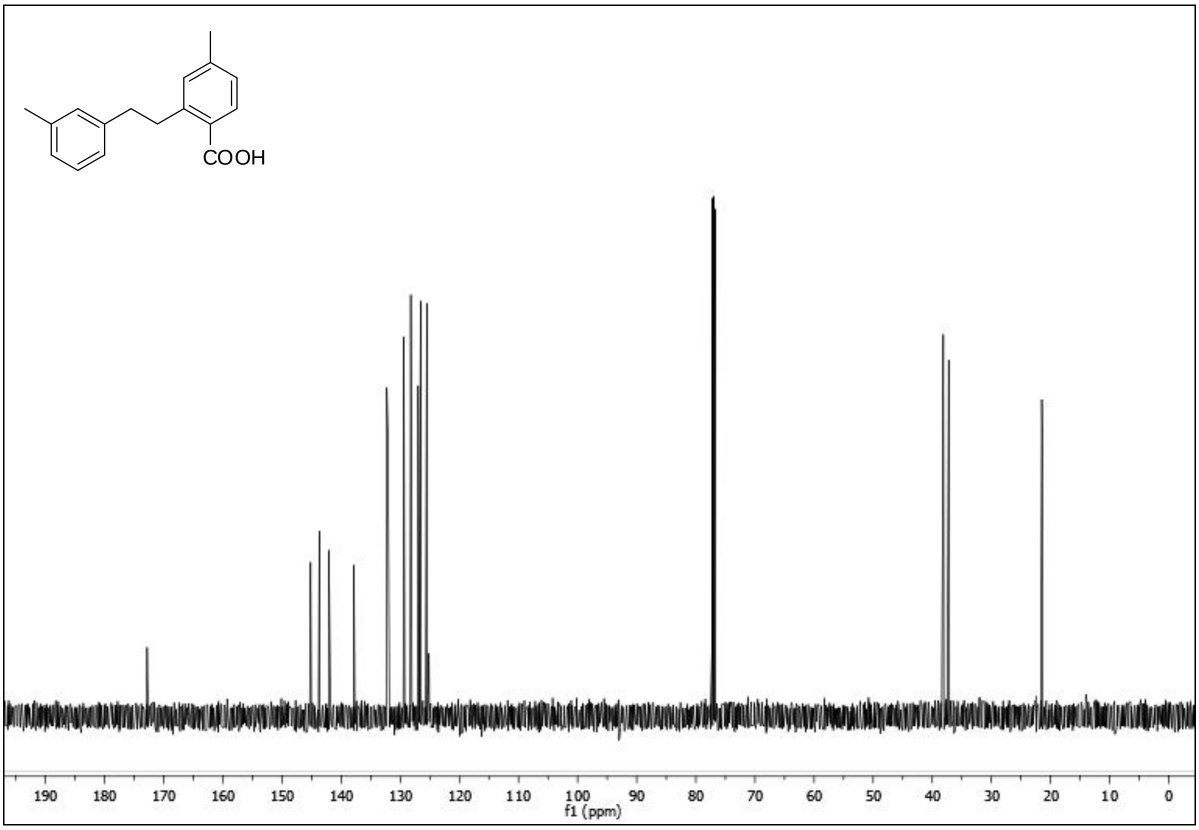

[2.2]Metacyclophane (8a).

^{1}H NMR

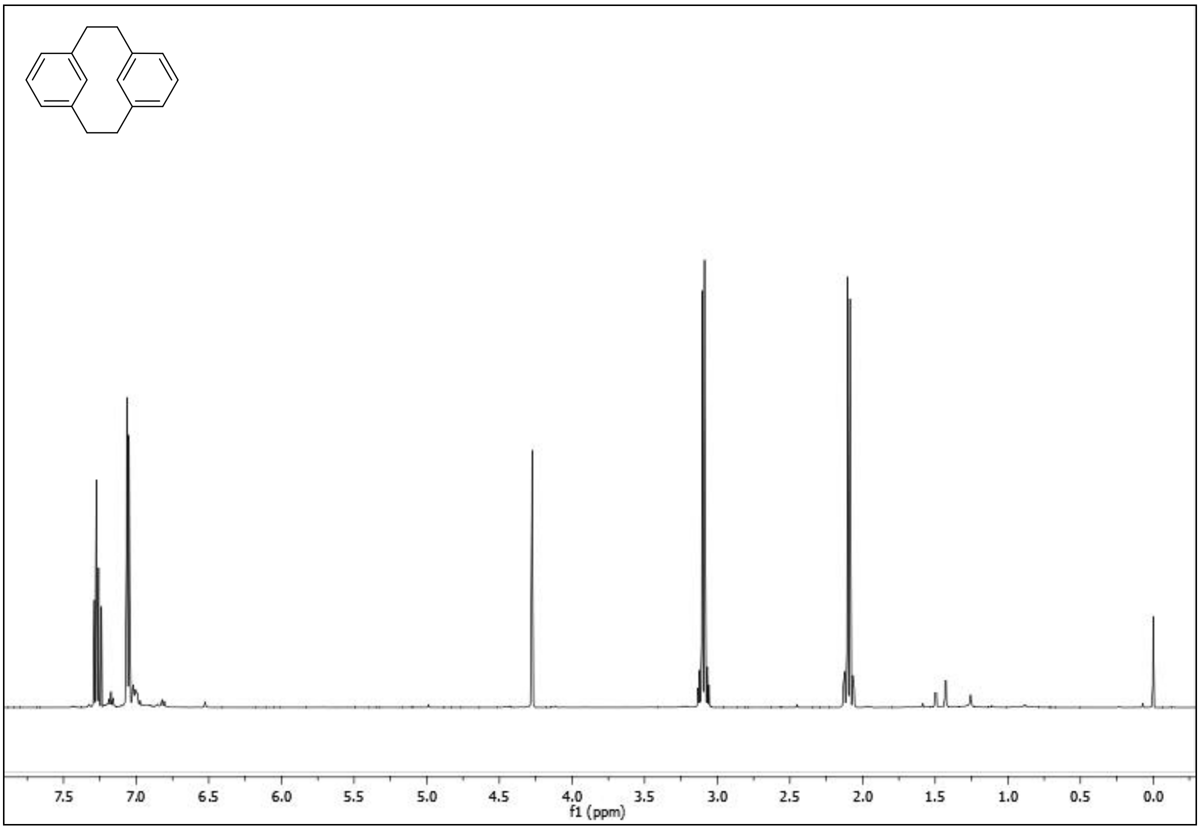

^{13}C NMR

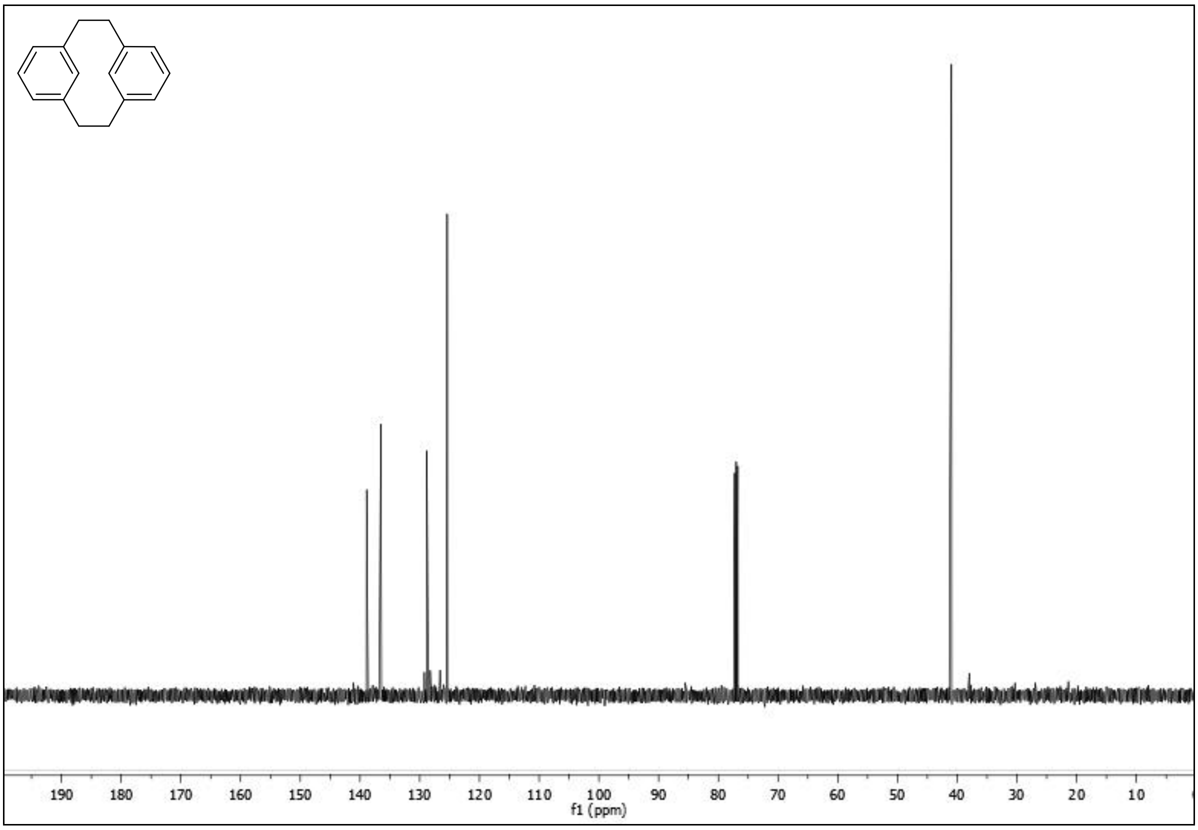

5,13-Dimethyl[2.2]metacyclophane (8b).

^{1}H NMR

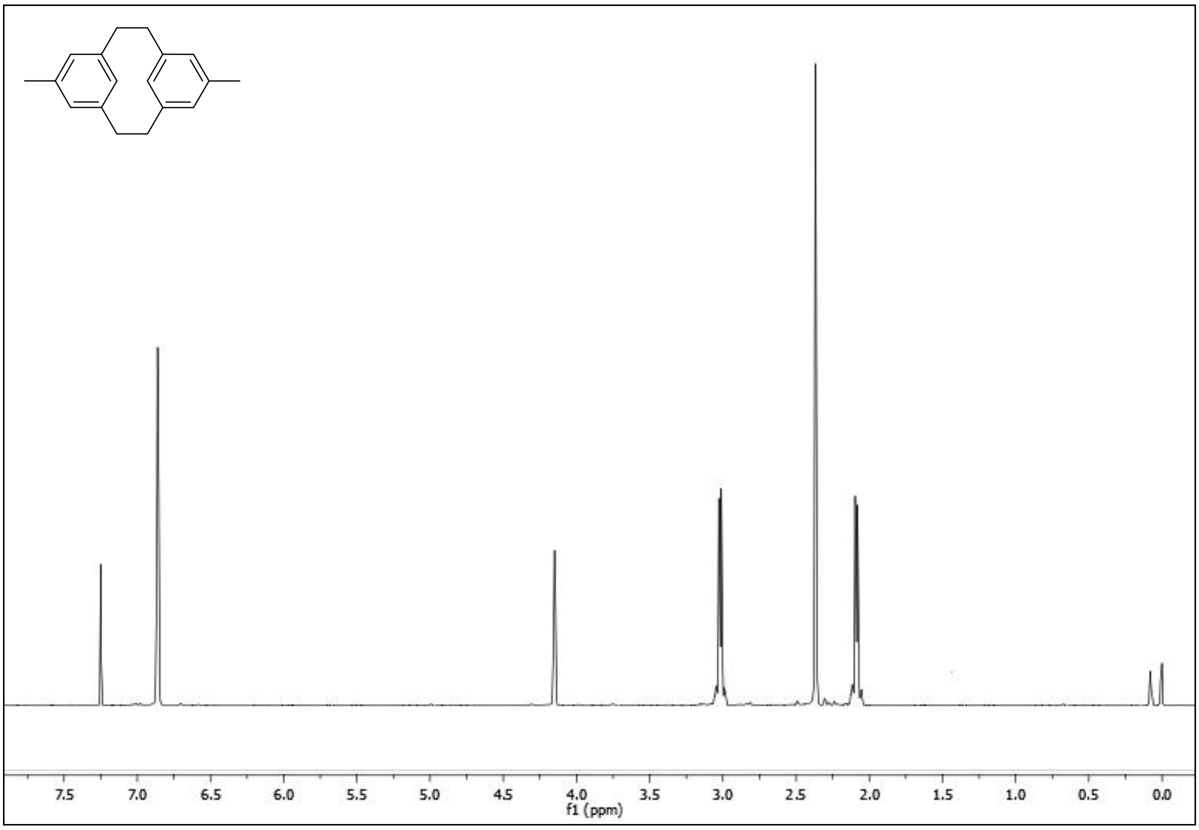

^{13}C NMR

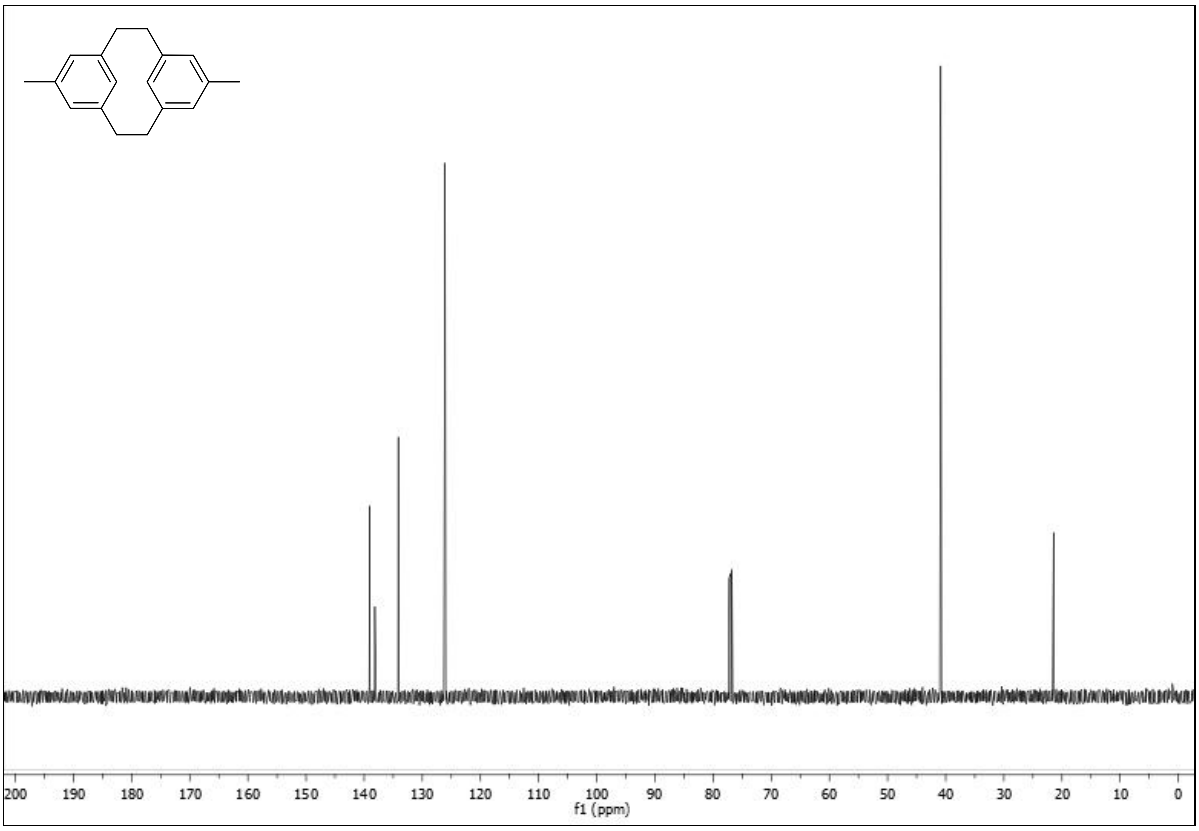

5,13-Dimethoxy[2.2]metacyclophane (8c).

^{1}H NMR

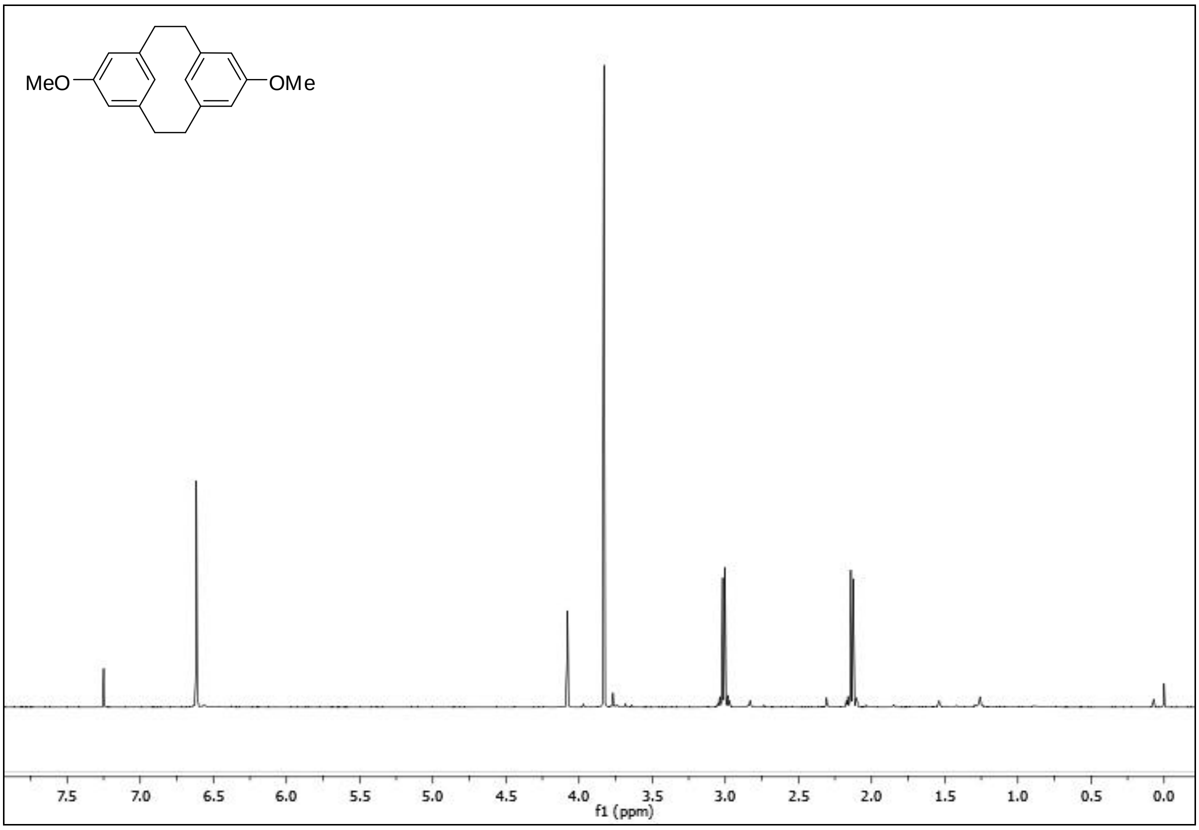

^{13}C NMR

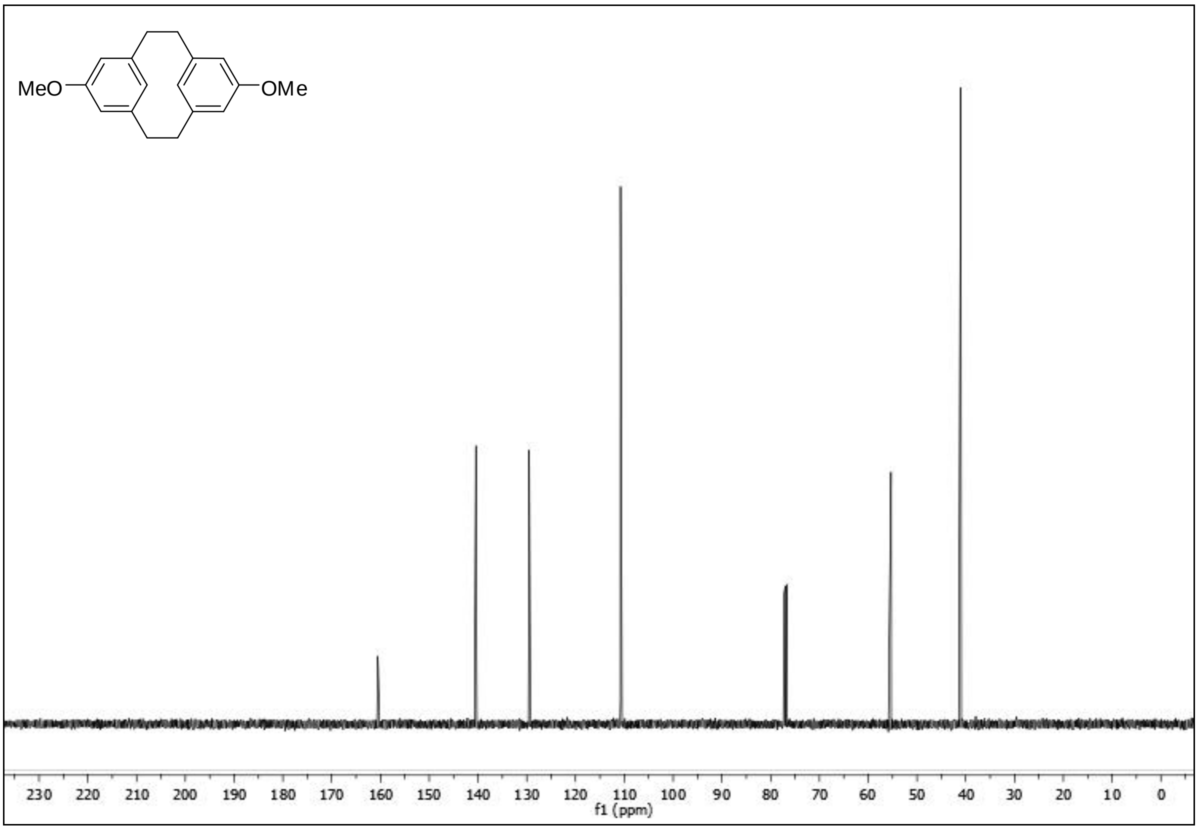

5,13-Bis(dimethylamino)[2.2]metacyclophane (8d).

^{1}H NMR

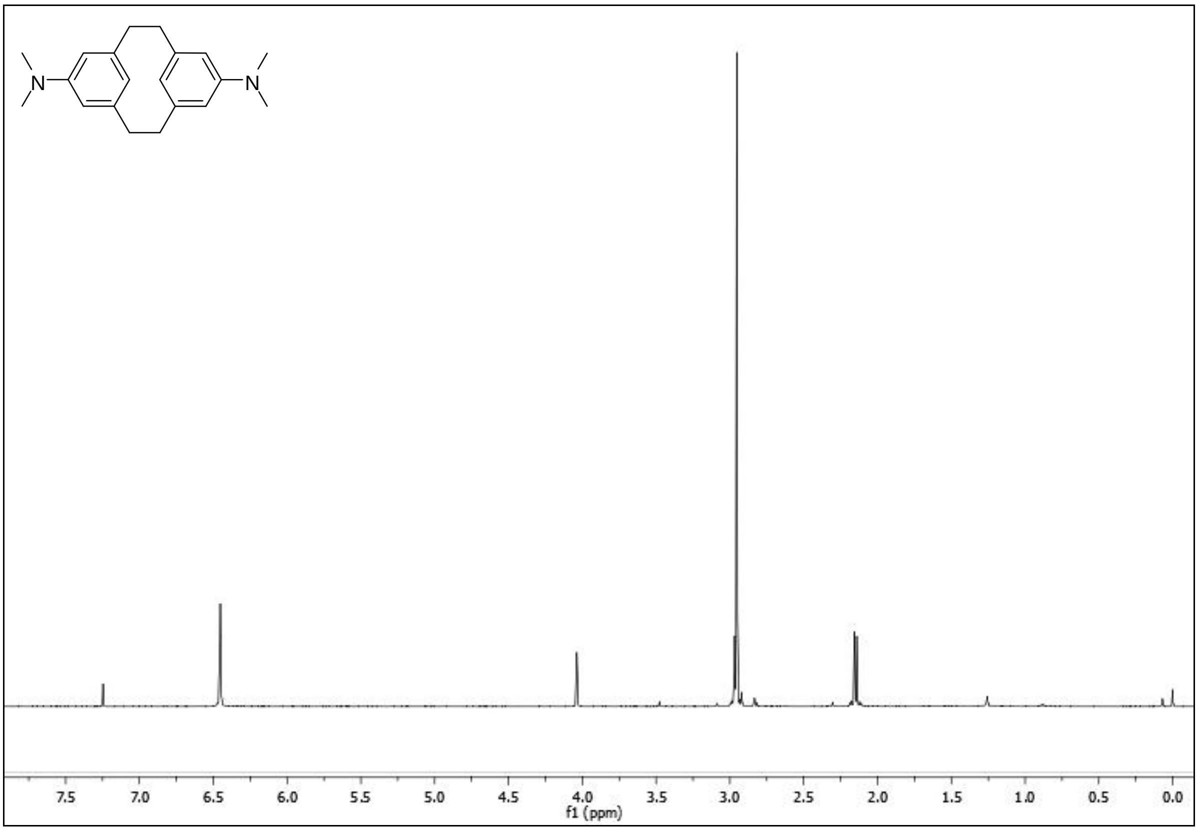

^{13}C NMR

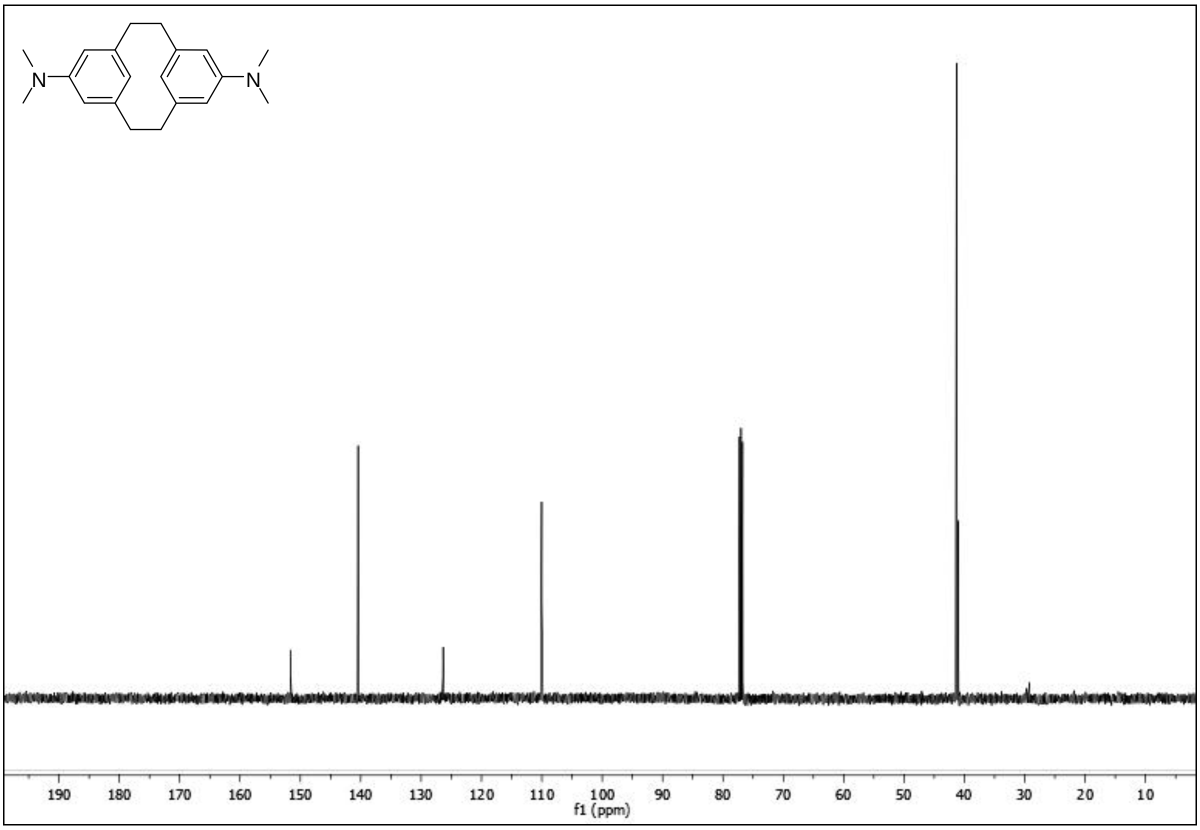

5-Methoxy[2.2]metacyclophane (8e).

^{1}H NMR

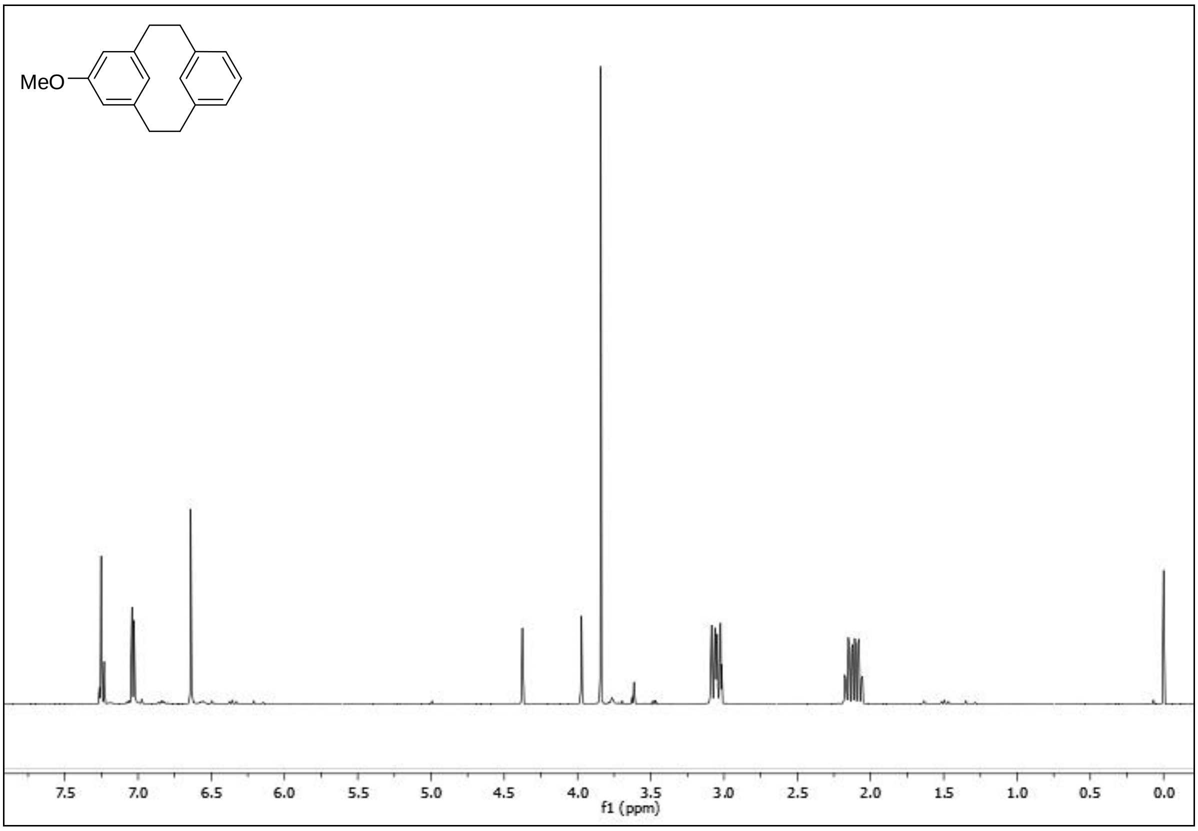

^{13}C NMR

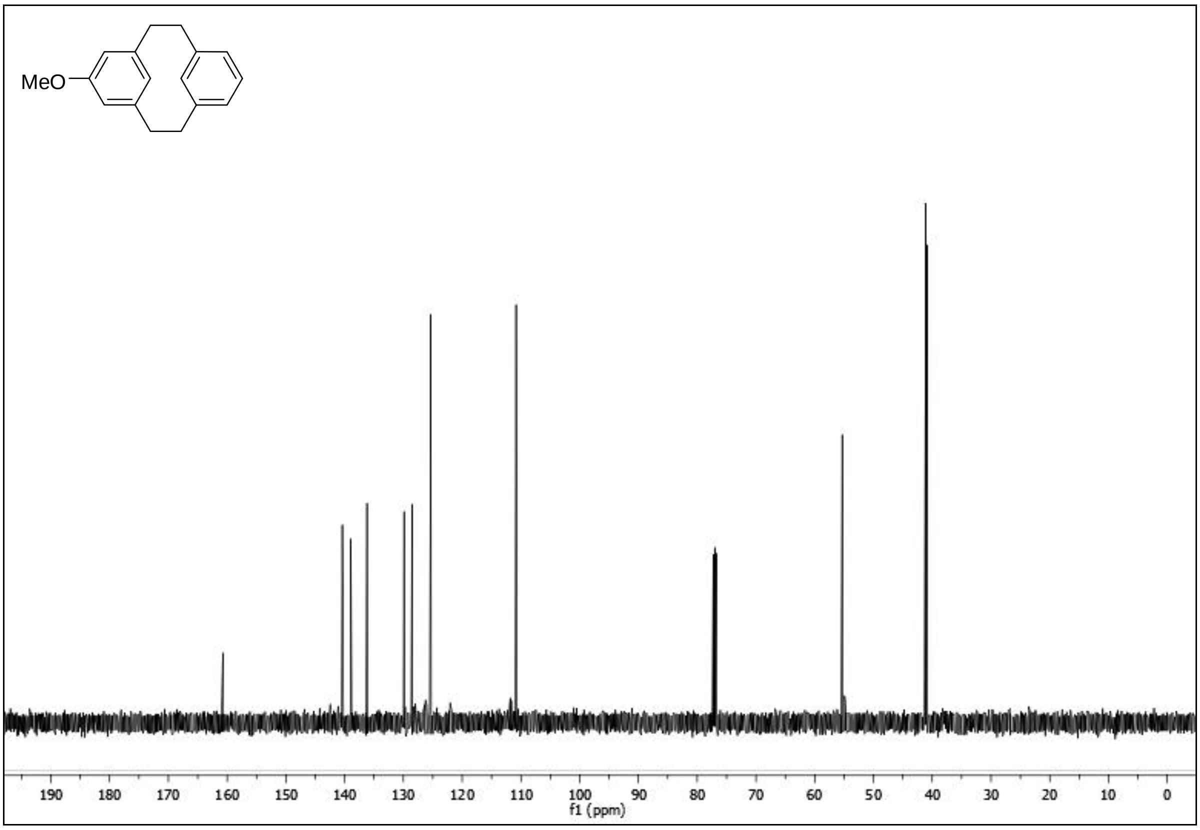

[2.2]metacyclophane-4-carboxylic acid (8f).

^{1}H NMR (CD_3OD)

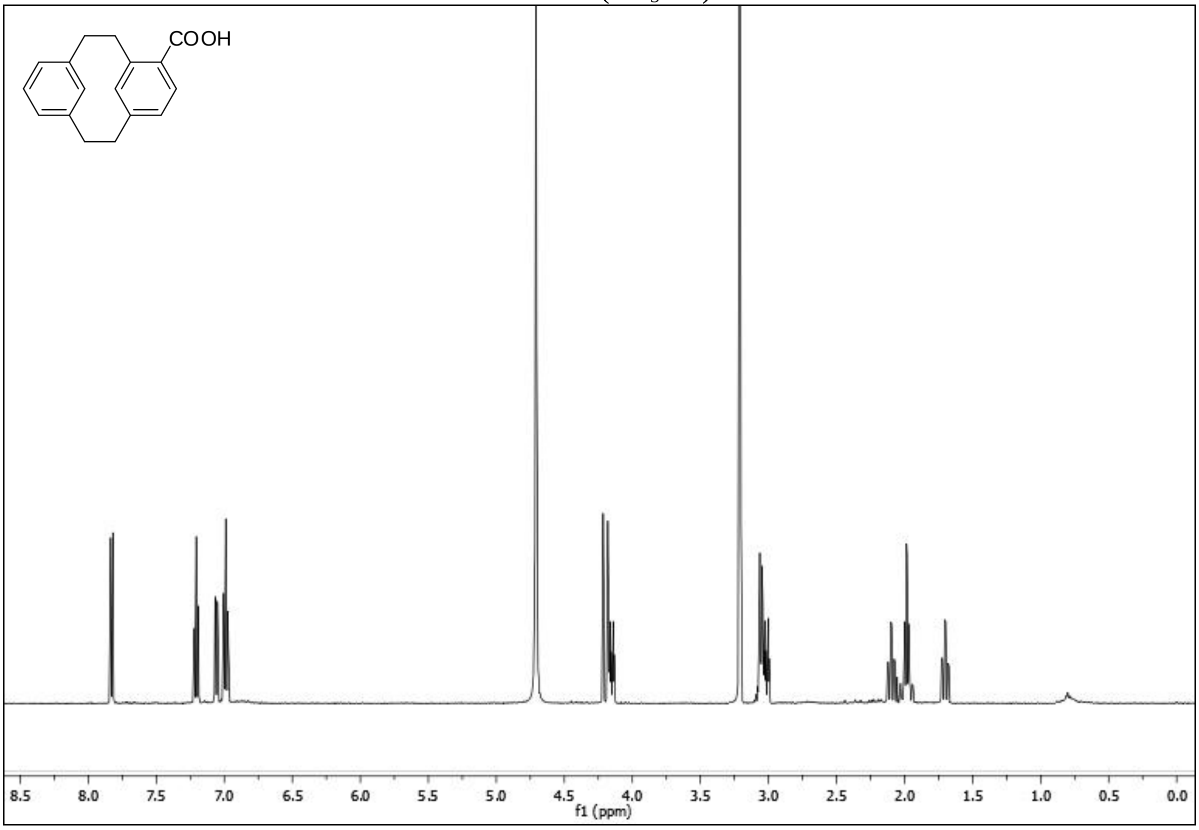

^{13}C NMR ($CDCl_3$)

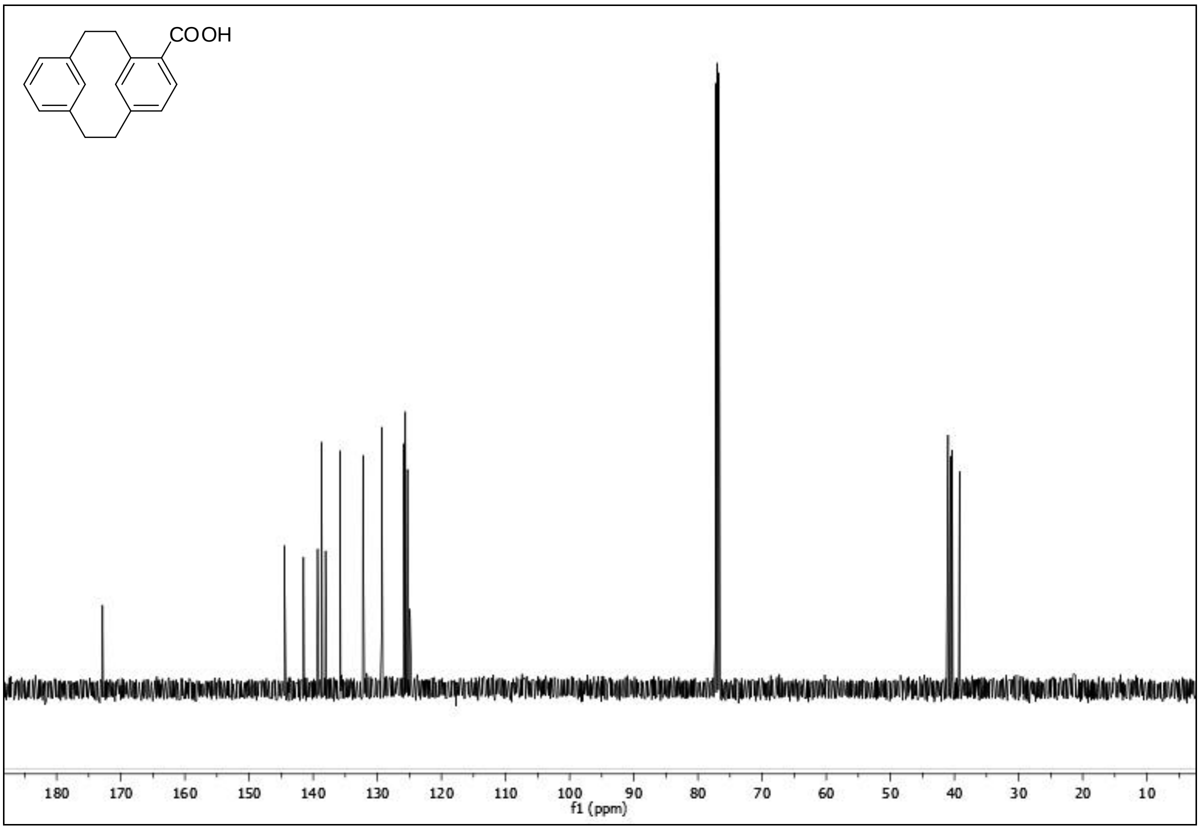

Synthesis and analysis of compounds D_1-**4e** and D_2-**6f**

2-(Deuteriomethyl)-4-methylbenzoic acid (D_1-4e). A solution of 2,4-dimethylbenzoic acid (**4e**) (75 mg, 0.50 mmol) in THF (10 mL) at –78 °C was treated dropwise with BuLi (2.50 M, 0.42 mL, 1.05 mmol) and stirred for 5 min. KO*t*-Bu (1.0 M in THF, 0.53 mL, 0.53 mmol) was added dropwise followed by 2,2,6,6-tetramethylpiperidine (84 µL, 0.50 mmol). The reaction mixture was stirred for 15 min at –78 °C and CD_3OD (60 µL) added. The reaction mixture was warmed to rt and the solvent removed under reduced pressure. Diethyl ether (20 mL) was added to the residue, washed with HCl (2 M, 3 x 10 mL), dried over sodium sulphate and concentrated to dryness to give **D_1-4e** as a white solid (69 mg, 91%). ^{1}H NMR (500 MHz, $CDCl_3$) δ 7.99 (d, J = 8.4 Hz, 1H), 7.09 (s, 2H), 2.63 (s, 2H), 2.38 (s, 3H); ^{13}C NMR (125 MHz, $CDCl_3$) δ 173.2, 143.6, 141.5, 132.7, 131.8, 126.6, 125.5, 22.1 (t, J = 20.0 Hz), 21.4; ^{2}H NMR (92.07 MHz, CH_2Cl_2) δ 2.63 (t, J = 2.1 Hz); HRMS $[M – H]^+$: 150.0658, $C_9H_8DO_2$ requires 150.0665.

4-(Deuteriomethyl)-2-(3-(deuteriomethyl)phenethyl)benzoic acid (D_2-6f). A solution of 4-methyl-2-(3-methylphenethyl)benzoic acid (**6f**) (50 mg, 0.20 mmol) in THF (7 mL) at –78 °C was treated dropwise with BuLi (2.50 M, 0.24 mL, 0.60 mmol) and stirred for 5 min. KO*t*-Bu (1.0 M in THF, 0.40 mL, 0.40 mmol) was added dropwise followed by 2,2,6,6-tetramethylpiperidine (68 µL, 0.40 mmol). The reaction mixture was stirred for 15 min at –78 °C and CD_3OD (60 µL) added. The reaction mixture was warmed to rt and the solvent removed under reduced pressure. Diethyl ether (20 mL) was added to the residue, washed with HCl (2 M, 3 x 10 mL), dried over sodium sulphate and concentrated to dryness to give **D_2-6f** as a pale yellow solid (45 mg, 87%). ^{1}H NMR (500 MHz, $CDCl_3$) δ 8.00 (d, J = 8.0 Hz, 1H), 7.17 (t, J = 7.5 Hz, 1H), 7.11 (d, J = 8.0 Hz, 1H), 7.09–7.03 (m, 3H), 7.00 (d, J = 7.5 Hz, 1H), 3.32–3.25 (m, 2H), 2.92–2.82 (m, 2H), 2.43–2.24 (m, 4H); ^{13}C NMR (125 MHz, $CDCl_3$) δ 172.8, 145.2, 143.7, 142.1, 137.8, 132.3, 132.0, 129.4, 128.2, 127.0, 126.6, 125.5, 38.1, 37.2, 21.3 (t, J = 19.3 Hz), 21.2 (t, J = 19.8 Hz); ^{2}H NMR (92.07 MHz, CH_2Cl_2) δ 2.44–2.18 (m); HRMS $[M – H]^+$: 255.1342, $C_{17}H_{15}D_2O_2$ requires 255.1354.

2-(Deuteriomethyl)-4-methylbenzoic acid (D_1-4e).

^{1}H NMR

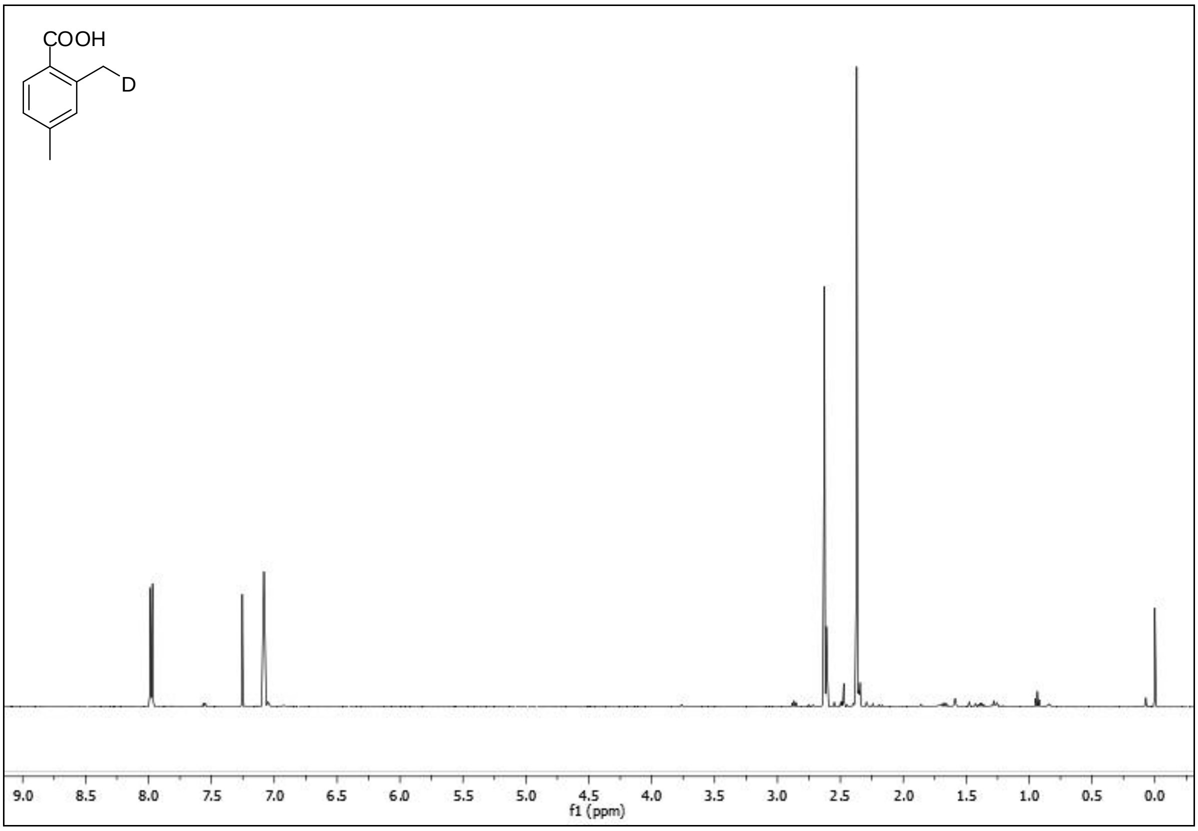

^{13}C NMR

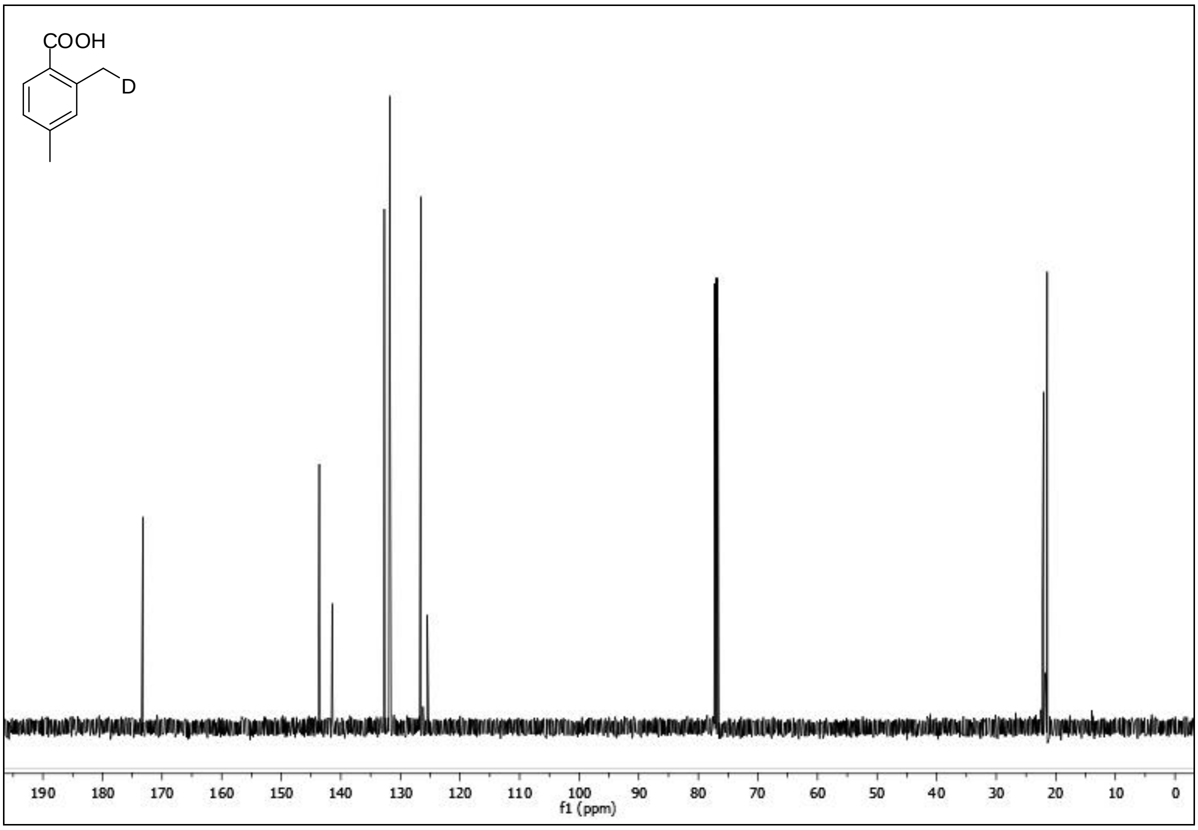

^{2}H NMR (CH_2Cl_2)

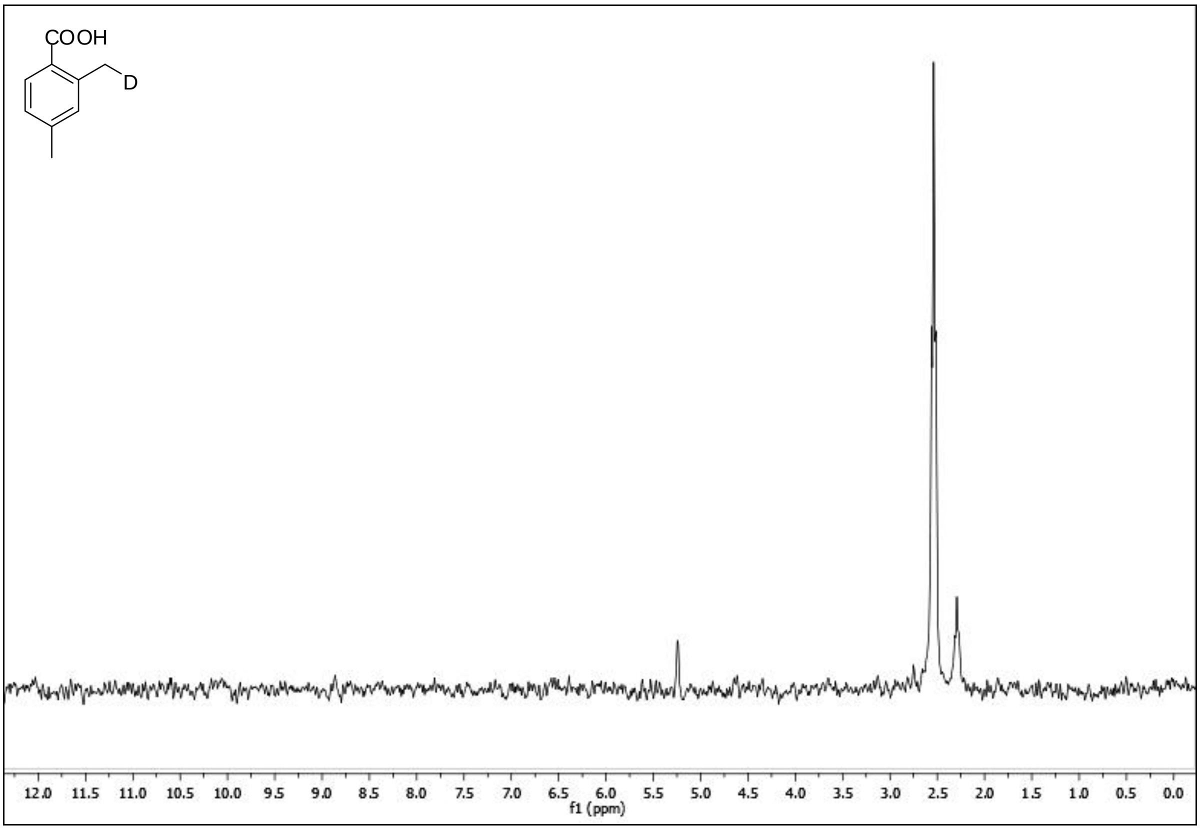

4-(Deuteriomethyl)-2-(3-(deuteriomethyl)phenethyl)benzoic acid (D_2-6f).

^{1}H NMR

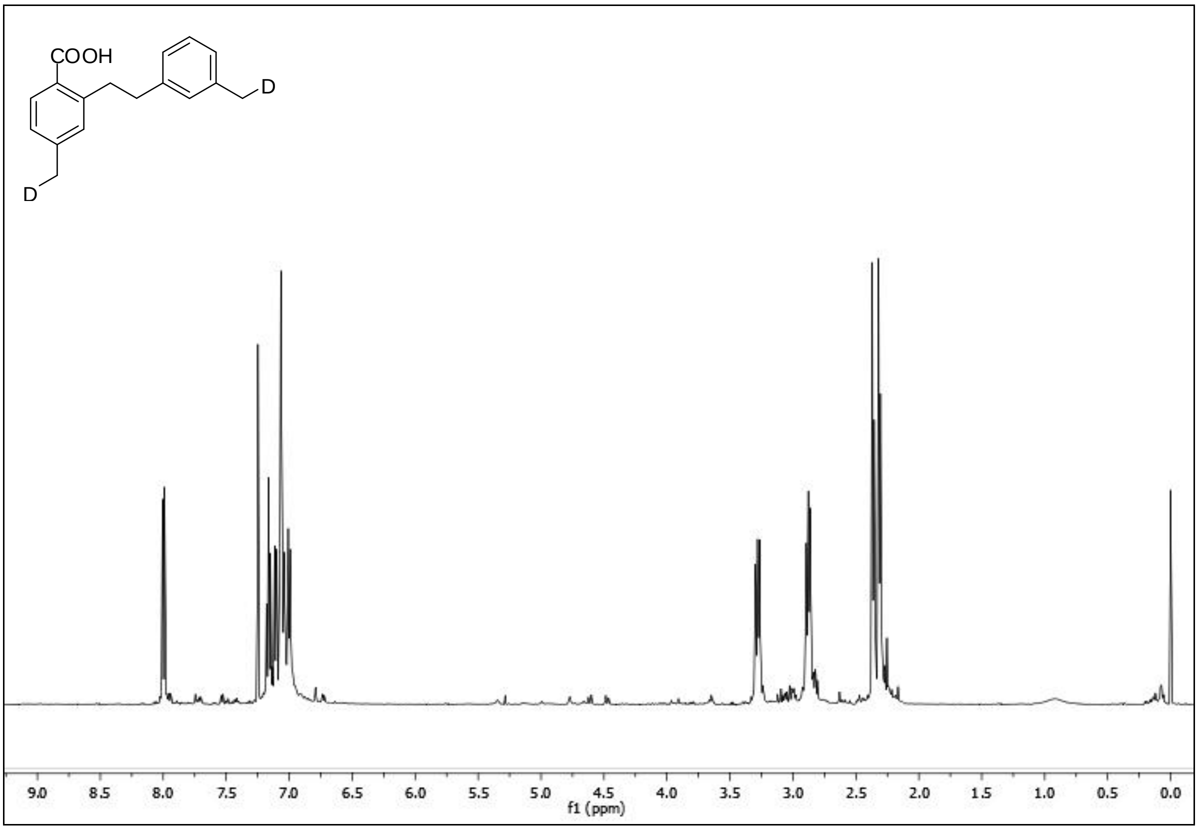

^{13}C NMR

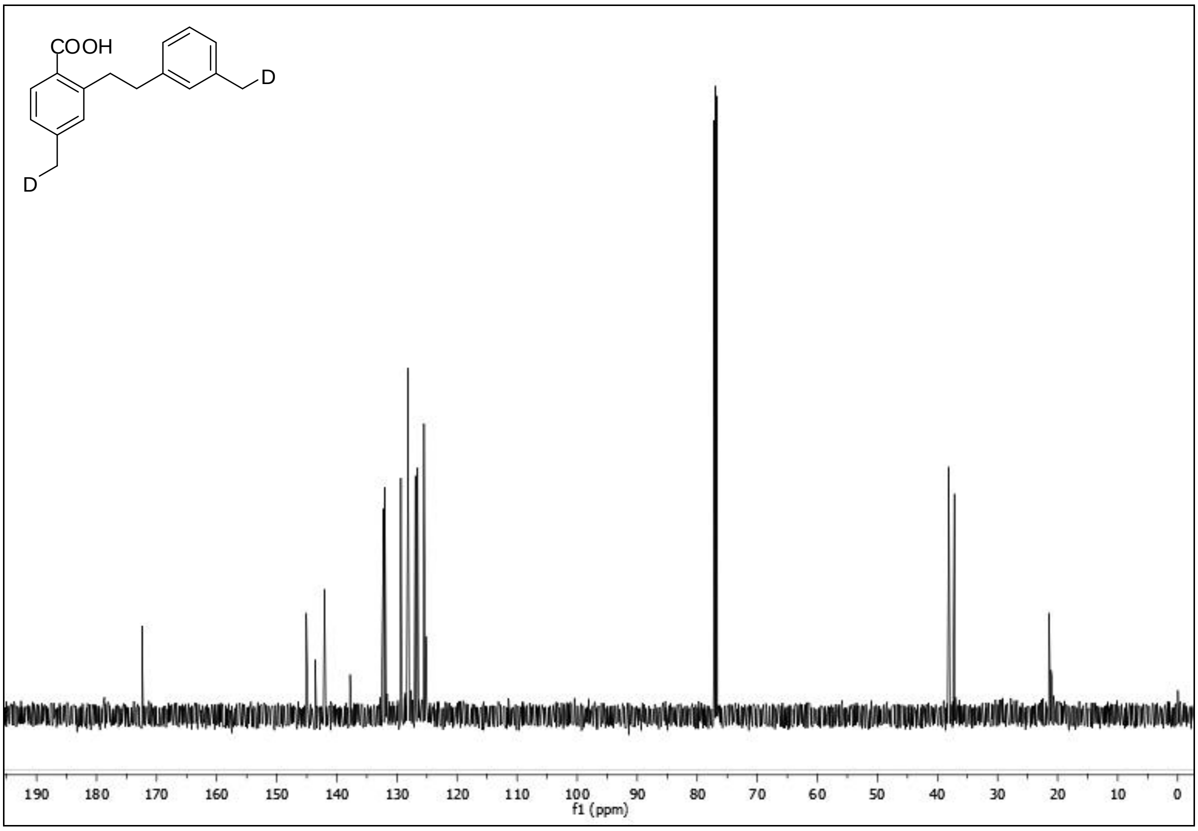

^2H NMR (CH_2Cl_2)

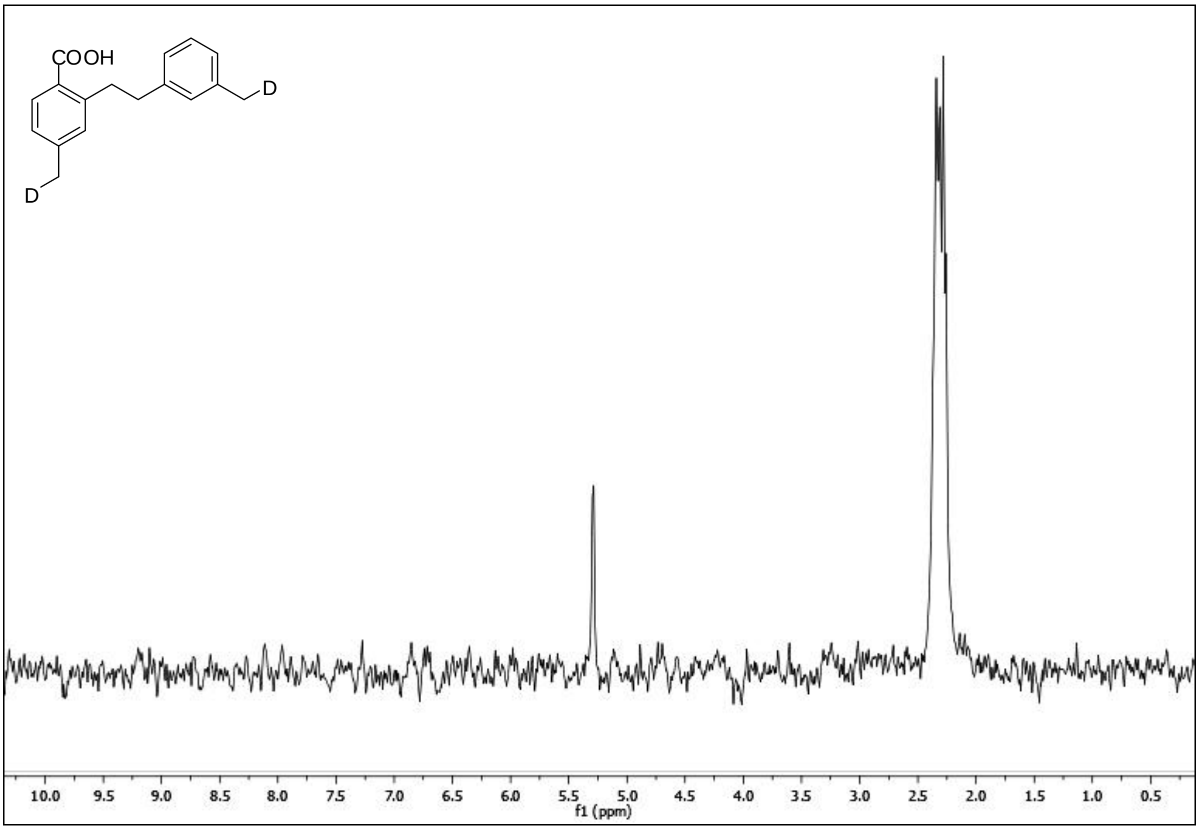

X-Ray Structural Data for **8c**

Table 1. Crystal data and structure refinement for **8c**.

Identification code	**8c**	
Empirical formula	$C_{18} H_{20} O_2$	
Formula weight	268.34	
Temperature	100(2) K	
Wavelength	1.54184 Å	
Crystal system	Monoclinic	
Space group	$P2_1/n$ (#14)	
Unit cell dimensions	a = 6.5997(1) Å	α = 90°.
	b = 8.9325(1) Å	β = 105.621(2)°.
	c = 12.2214(2) Å	γ = 90°.
Volume	693.862(19) $Å^3$	
Z	2	
Density (calculated)	1.284 Mg/m^3	
Absorption coefficient	0.645 mm^{-1}	
F(000)	288	
Crystal size	0.2018 x 0.0769 x 0.0564 mm^3	
Theta range for data collection	6.22 to 76.95°.	
Index ranges	–8<=h<=8, –11<=k<=11, –15<=l<=15	
Reflections collected	9820	
Independent reflections	1453 [R(int) = 0.0284]	
Completeness to theta = 76.95°	99.2 %	
Absorption correction	Analytical	
Max. and min. transmission	0.969 and 0.904	
Refinement method	Full–matrix least–squares on F^2	
Data / restraints / parameters	1453 / 0 / 132	
Goodness–of–fit on F^2	1.069	
Final R indices [I>2sigma(I)]	R1 = 0.0310, wR2 = 0.0805	
R indices (all data)	R1 = 0.0342, wR2 = 0.0830	
Extinction coefficient	0.0062(11)	
Largest diff. peak and hole	0.250 and –0.163 e.$Å^{-3}$	

Table 2. Atomic coordinates (x 10^4) and equivalent isotropic displacement parameters (Å^{2}x 10^3) for **8c**. U(eq) is defined as one third of the trace of the orthogonalized U^{ij} tensor.

Atom	x	Y	z	U(eq)
C(1)	10586(2)	10321(1)	7462(1)	18(1)
O	11137(1)	9984(1)	6484(1)	22(1)
C(7)	12811(2)	10827(1)	6252(1)	24(1)
C(2)	11702(1)	11300(1)	8298(1)	18(1)
C(3)	10924(2)	11639(1)	9226(1)	18(1)
C(4)	9001(1)	11037(1)	9272(1)	18(1)
C(5)	8034(1)	9901(1)	8522(1)	18(1)
C(8)	6490(1)	8873(1)	8862(1)	20(1)
C(9)	7665(2)	7647(1)	9721(1)	20(1)
C(6)	8818(2)	9563(1)	7604(1)	19(1)

Table 3. Bond lengths [Å] and angles [°] for **8c**.

C(1)–O	1.3727(12)
C(1)–C(2)	1.3950(14)
C(1)–C(6)	1.3997(14)
O–C(7)	1.4266(12)
C(7)–H(7A)	0.978(15)
C(7)–H(7B)	0.990(14)
C(7)–H(7C)	0.988(14)
C(2)–C(3)	1.3985(14)
C(2)–H(2)	0.983(13)
C(3)–C(4)	1.3939(13)
C(3)–C(9)#1	1.5115(13)
C(4)–C(5)	1.4008(13)
C(4)–H(4)	0.965(12)
C(5)–C(6)	1.3900(13)
C(5)–C(8)	1.5104(13)
C(8)–C(9)	1.5698(14)
C(8)–H(8A)	1.009(13)
C(8)–H(8B)	0.988(14)
C(9)–C(3)#1	1.5115(13)
C(9)–H(9A)	0.987(13)
C(9)–H(9B)	0.994(13)
C(6)–H(6)	0.973(14)
O–C(1)–C(2)	124.31(9)
O–C(1)–C(6)	115.24(9)
C(2)–C(1)–C(6)	120.41(9)
C(1)–O–C(7)	117.24(8)
O–C(7)–H(7A)	111.2(8)
O–C(7)–H(7B)	105.5(8)
H(7A)–C(7)–H(7B)	109.5(11)

O–C(7)–H(7C)	110.4(8)
H(7A)–C(7)–H(7C)	110.2(11)
H(7B)–C(7)–H(7C)	109.9(11)
C(1)–C(2)–C(3)	119.53(9)
C(1)–C(2)–H(2)	120.2(8)
C(3)–C(2)–H(2)	120.3(8)
C(4)–C(3)–C(2)	119.13(9)
C(4)–C(3)–C(9)#1	119.16(8)
C(2)–C(3)–C(9)#1	120.37(9)
C(3)–C(4)–C(5)	120.81(9)
C(3)–C(4)–H(4)	120.2(7)
C(5)–C(4)–H(4)	118.3(7)
C(6)–C(5)–C(4)	118.88(9)
C(6)–C(5)–C(8)	121.03(9)
C(4)–C(5)–C(8)	118.84(8)
C(5)–C(8)–C(9)	111.07(7)
C(5)–C(8)–H(8A)	110.7(8)
C(9)–C(8)–H(8A)	108.3(7)
C(5)–C(8)–H(8B)	110.1(8)
C(9)–C(8)–H(8B)	108.3(8)
H(8A)–C(8)–H(8B)	108.2(11)
C(3)#1–C(9)–C(8)	110.78(8)
C(3)#1–C(9)–H(9A)	111.3(7)
C(8)–C(9)–H(9A)	107.4(7)
C(3)#1–C(9)–H(9B)	110.6(7)
C(8)–C(9)–H(9B)	108.2(7)
H(9A)–C(9)–H(9B)	108.4(10)
C(5)–C(6)–C(1)	119.92(9)
C(5)–C(6)–H(6)	121.0(8)
C(1)–C(6)–H(6)	119.0(7)

Symmetry transformations used to generate equivalent atoms:
#1 –x+2,–y+2,–z+2

Table 4. Anisotropic displacement parameters ($Å^2 x 10^3$) for **8c**. The anisotropic displacement factor exponent takes the form: $-2\pi^2[h^2 a^{*2}U^{11} + ... + 2 h k a^* b^* U^{12}]$

Atom	U^{11}	U^{22}	U^{33}	U^{23}	U^{13}	U^{12}
C(1)	20(1)	19(1)	16(1)	3(1)	5(1)	4(1)
O	25(1)	24(1)	19(1)	–1(1)	10(1)	–3(1)
C(7)	23(1)	28(1)	23(1)	2(1)	10(1)	–1(1)
C(2)	17(1)	18(1)	19(1)	4(1)	4(1)	1(1)
C(3)	19(1)	14(1)	18(1)	2(1)	3(1)	1(1)
C(4)	18(1)	17(1)	17(1)	1(1)	4(1)	3(1)
C(5)	15(1)	19(1)	17(1)	3(1)	2(1)	2(1)
C(8)	17(1)	23(1)	18(1)	–1(1)	3(1)	–3(1)
C(9)	21(1)	19(1)	19(1)	–1(1)	6(1)	–4(1)
C(6)	19(1)	19(1)	16(1)	0(1)	2(1)	0(1)

Table 5. Hydrogen coordinates (x 10^4) and isotropic displacement parameters ($Å^2$x 10^3) for **8c**.

Atom	x	y	z	U(eq)
H(7A)	14170(20)	10552(16)	6771(12)	31(3)
H(7B)	12800(20)	10564(16)	5464(12)	29(3)
H(7C)	12560(20)	11911(16)	6306(11)	28(3)
H(2)	13030(20)	11742(15)	8237(11)	24(3)
H(4)	8424(19)	11279(14)	9897(10)	18(3)
H(8A)	5540(20)	9457(15)	9233(10)	22(3)
H(8B)	5600(20)	8363(15)	8185(12)	27(3)
H(9A)	6582(19)	7011(14)	9909(10)	20(3)
H(9B)	8501(19)	7018(15)	9332(11)	23(3)
H(6)	8180(20)	8784(15)	7064(11)	25(3)

Table 6. Torsion angles [°] for **8c.**

C(2)–C(1)–O–C(7)	–7.73(13)
C(6)–C(1)–O–C(7)	174.51(8)
O–C(1)–C(2)–C(3)	175.86(8)
C(6)–C(1)–C(2)–C(3)	–6.48(14)
C(1)–C(2)–C(3)–C(4)	–2.64(14)
C(1)–C(2)–C(3)–C(9)#1	164.04(9)
C(2)–C(3)–C(4)–C(5)	11.60(14)
C(9)#1–C(3)–C(4)–C(5)	–155.24(9)
C(3)–C(4)–C(5)–C(6)	–11.29(14)
C(3)–C(4)–C(5)–C(8)	156.10(9)
C(6)–C(5)–C(8)–C(9)	88.51(10)
C(4)–C(5)–C(8)–C(9)	–78.59(11)
C(5)–C(8)–C(9)–C(3)#1	58.44(10)
C(4)–C(5)–C(6)–C(1)	2.02(14)
C(8)–C(5)–C(6)–C(1)	–165.08(9)
O–C(1)–C(6)–C(5)	–175.34(8)
C(2)–C(1)–C(6)–C(5)	6.80(14)

Symmetry transformations used to generate equivalent atoms:

#1 –x+2,–y+2,–z+2
